# How well do large language models mirror human cognition of word concepts?: A comparison of psychological ratings for early-acquired English words

**DOI:** 10.3758/s13428-025-02938-2

**Published:** 2026-02-02

**Authors:** Hiromichi Hagihara, Kazuki Miyazawa

**Affiliations:** 1 Graduate School of Human Sciences, The University of Osaka, 1-2 Yamadaoka, Suita-shi, Osaka, 565-0871 Japan; 2 International Research Center for Neurointelligence (WPI-IRCN), Institutes for Advanced Study, The University of Tokyo, 7-3-1 Hongo, Bunkyo-ku, Tokyo, 113-0033 Japan; 3 Graduate School of Engineering Science, The University of Osaka, 1-3 Machikaneyama, Toyonaka-shi, Osaka, 560-8531 Japan

**Keywords:** Large language model, Lexical psychological feature, Language development, Age of acquisition, Automated rating, Machine psychology

## Abstract

This study examined how well large language models (LLMs) approximate human psychological ratings for early-acquired English words. We used four state-of-the-art LLMs, including GPT-4o and Meta-Llama-3.1, to evaluate 21 static psychological features for 695 words and compared these estimates with human norms. The results showed that LLMs aligned well with human ratings for some features (e.g., Concreteness, Bodily Interactiveness) in terms of rank correlations (*r*s > .82) and distributional similarities but diverged notably for others (e.g., Iconicity, Arousal; *r*s < .48). Compared with content words, function words showed more pronounced discrepancies between human and LLM ratings. We also assessed how similarly human- and LLM-derived psychological features predicted words’ age of acquisition (AoA), revealing both strong correspondences and systematic biases, depending on the model (differences in correlations ranged from −.27 to .28). Based on these analyses, we identified which features may be reliably estimated using LLMs, which require further refinement, and what methodological considerations are necessary for applying LLM-based measures in cognitive science. We discuss the implications of using LLMs as methodological tools in psychology and cognitive science, highlighting both their practical advantages (e.g., data coverage and data collection efficiency) and theoretical relevance. The present study provides a novel framework for evaluating the cognitive plausibility of LLMs by using lexical psychological features, complementing existing benchmarks.

**Supplementary Information:**

The online version contains supplementary material available at 10.3758/s13428-025-02938-2.

Recent large language models (LLMs), trained on vast datasets, exhibit remarkable proficiency across a wide range of tasks (Frank, 2023; Teubner et al., 2023). LLMs have been applied in the real world; for example, in brainstorming ideas, proofreading and grammar checking, coding assistance, and translation. LLMs have also been leveraged for specific research purposes in the field of psychology and cognitive science (for reviews see Demszky et al., 2023; Ke et al., 2024): How different are datasets and learning processes between LLMs and human children (Feng et al., 2024; Frank, 2023)? How can LLMs support clinical assessments and interventions (Schueller & Morris, 2023; Singhal et al., 2023)? How much human intelligence is embedded in LLMs (Marjieh et al., 2024; Strachan et al., 2024)? In particular, some researchers have suggested “machine psychology,” which investigates and assesses LLMs’ behavioral patterns and emergent abilities by regarding them as participants in psychological experiments (Binz & Schulz, 2023; Frank & Goodman, 2025; Hagendorff et al., 2024). Relatedly, Lin (2025) proposed a broader framework for using LLMs as psychological simulators, highlighting their potential role in modeling human behavior across cognitive domains. Given that the huge amount of highly compressed language data may contain key aspects of human behavior and cognition, extracting such information from LLMs as a new approach in psychology and cognitive science can help in understanding the features and proficiency of LLMs as well as human minds (but see Stokel-Walker, 2024).

Such LLMs’ *cognitive* abilities may be useful for advancing scientific research more efficiently. Recent research on psycholinguistics and language development has actively utilized existing datasets containing various static, word-level psychological features rated by human participants to examine how word concepts interrelate with each other (Diveica et al., 2024, 2025) or what psychological features make words easier for young children to learn (Tan et al., 2024). There are many different kinds of available measurements that human participants have rated with Likert scales for each word, including concreteness (Brysbaert et al., 2014), sensory modality (Lynott et al., 2020), socialness (Diveica et al., 2022), and arousal (Warriner et al., 2013). For instance, “peach” is very concrete, physically interactable, and relevant to specific sensory modalities such as touch, smell, or taste, while “peace” is very abstract, only socially or linguistically explainable, and amodal, thus assumed to be hard for young children to learn.

However, because different studies have examined different psychological features for different sets of target words, the resulting data are incomplete; some words hold certain features and lack others. This has challenged researchers to comprehensively investigate word concepts. Researchers may also face difficulties in developing new context-independent psychological features because new data collection for a vast set of target words is time-consuming and costly. Given the recent proficiency of LLMs’ cognitive abilities, though relying solely on linguistic knowledge without a body, it may be possible for them to reproduce a wide range of lexical psychological features that resemble human participants. Recent work has begun to explore this possibility for existing English norms (e.g., Trott, 2024). If this possibility is the case, it opens up new opportunities to prepare complete (without missing) datasets of existing psychological features or develop new psychological feature ratings using LLMs as a replacement for human participants, although some validation checks may be required (Hämäläinen et al., 2023; see also Conde et al., 2025, for a practical guide).

In the present study, we specifically focused on English words that are acquired early by young children, used several state-of-the-art LLMs to evaluate diverse psychological features, and analyzed the similarities and differences in ratings between LLMs and human adults. We focused on early-acquired words for two reasons. First, because many early-acquired words rely more on physical or social knowledge (i.e., *world knowledge*) than on purely linguistic knowledge (i.e., *word knowledge*) (Diveica et al., 2025; Muraki et al., 2022; Tan et al., 2024), the differences in cognition between LLMs and humans are expected to be more clearly observable. Second, in addition to examining the similarities and differences in lexical psychological features between LLMs and humans, limiting the targeted words to early-acquired words allowed us to evaluate the performance of LLM-generated ratings in relation to *development* as an external factor. Accumulated findings exist in language development and psycholinguistic research on the relationships between different psychological features and the ease or difficulty of acquiring a word, serving as a reference (e.g., Diveica et al., 2025).

Examining the resemblance of lexical psychological features between LLMs and human participants provides a foundation for broadening the scope of research, both theoretically and methodologically. For example, if we find certain features that are very similar between machines and humans, the same method could potentially be applied to other languages. Although this study is limited to English, where both LLM performance and human norm data are relatively well established, it represents a necessary first step in evaluating whether LLM-derived psychological ratings can approximate human judgments across languages. This direction is particularly valuable for research on language development, given that psychological features in non-English languages are either scarcely available or have not been studied, despite the existence of large open datasets on developmental aspects (e.g., age of acquisition: AoA) for at least several hundred words in more than 40 languages (Frank et al., 2017). Recent work suggests that LLMs can function effectively across many languages, and multilingual models offer a promising direction (e.g., Ahuja et al., 2023; Peng et al., 2025; Wang et al., 2024; see Qin et al., 2025, for a review). In line with this, initial attempts have been made to predict concreteness ratings across multiple languages using transformer-based models (Kewenig et al., 2025), suggesting that certain psychological features may generalize beyond English. Leveraging LLMs may thus serve as a first step toward comprehensively estimating lexical psychological features in a wide range of languages.

In this study, we used several state-of-the-art LLMs to evaluate 21 static psychological features of 695 English words acquired early in life by young children. We had three purposes. First, we examined the extent to which lexical psychological features estimated by state-of-the-art LLMs resemble those estimated by humans. Second, we examined how similar or different the predictability of each psychological feature for lexical development (i.e., AoA) as an external factor was between the LLMs’ ratings and human ratings. Third, based on these comparisons, we organized promising features by which LLMs could be used as replacements for human participants, features that still need improvement, and points to consider when using LLM-evaluated lexical psychological features in cognitive science. Our prompts, codes, and exported ratings estimated using the LLMs can be found at https://github.com/hagi-hara/psychfeaturesllm.

## Methods

### Targeted words

We selected a set of target words based on the MacArthur-Bates Communicative Development Inventories (CDI: Fenson et al., 1993), the most widely used parent-report vocabulary measurement in language development research. As some of the words in the CDI included two or more words in the same item (e.g., “soda/pop,” “tissue/Kleenex,” “inside/in”), we divided those words and used them separately in this study. Some word items listed in the plural form (e.g., “bubbles,” “carrots”) were changed to the singular form. We used polysemous words and homophones as they were listed in the CDI, and if supplementary information was included in the item (e.g., “chicken (animal)” versus “chicken (food),” “drink (beverage)” versus “drink (action)”), we included it when asking LLMs to estimate lexical psychological features. However, it should be noted that the existing datasets of psychological features rated by human participants virtually did not distinguish between the two meanings of polysemous words and homophones, and thus the same rating values were used for both meanings. The occurrence of such atypical word items accounted for only approximately 2–6% of the total (see Supplementary Table S1), suggesting that their influence on the overall results is minimal. The final list of our target words contained 695 words.

We calculated each word's age of acquisition (AoA), a typical measure of children's lexical development, using Wordbank (Frank et al., 2017), an open repository of CDI data (accessed on May 19, 2024). We fitted logistic regressions for each word using *wordbankr* (Braginsky, 2024) to obtain AoAs, the age in months at which 50% of the children are predicted to produce the word.

### Human datasets

Based on previous research that explored the relationships among various lexical psychological features (e.g., Diveica et al., 2024) and the predictability of young children's lexical development (e.g., Campbell et al., 2024; Tan et al., 2024), we selected the features to be used in this study. Additionally, we considered whether adult norms and instructions for participants were available. Finally, 21 indices were selected. All indices were quantified by asking adult participants to rate the psychological properties of English words on a 5- to 10-point Likert scale.

- *Concreteness* (Brysbaert et al., 2014): The extent to which words are concrete, meaning that they are grounded in sensory and motor experiences versus abstract, relying primarily on linguistic representations.
- *Imageability* (Cortese & Fugett, 2004; Schock et al., 2012): The extent to which words arouse a sensory experience (e.g., mental image or sound) quickly and easily. It is known that imageability correlates with concreteness.
- *Bodily interactiveness* (Muraki et al., 2022; Pexman et al., 2019): How easily the human body can interact with a referent of a word physically, which is called body–object interaction (BOI). There are two measures: Adult BOI and Child BOI. For instance, although “fire” is physically interactable, its Child BOI is low, as fire is dangerous and thus difficult for children to interact with bodily.
- *Iconicity* (Winter et al., 2023): The extent to which a word form (i.e., label) is perceived as similar to its meaning. High iconicity has high form-meaning similarity (e.g., “zigzag”), while low iconicity indicates that the relationship between word form and its meaning seems rather arbitrary (e.g., “menu”).
- *Socialness* (Diveica et al., 2022): The degree to which a word has social relevance, such as the social characteristic of a person or group (e.g., “police”), social role (e.g., “friend”), or social behavior or interaction (e.g., “share”).
- *Babiness* (Perry et al., 2015): The degree to which a word is associated with infants. Highly rated words include “cute,” “milk,” and “peekaboo.”
- *Valence, Arousal, and Dominance* (Warriner et al., 2013): The extent to which words invoke three different types of emotions. Valence is a rating of how happy or pleasant a word makes a person feel (e.g., “hug” or “love”). Arousal refers to the intensity of emotion provoked by a word (e.g., “hate” or “party”). Dominance indicates the degree to which a word makes a person feel in control or powerful, as opposed to submissive or weak (e.g., “smile” or “successful”).
- *Sensorimotor strength* (Lynott et al., 2020): The extent to which a word evokes sensorimotor strength across six perceptual modalities (Auditory, Gustatory, Haptic, Interoceptive, Olfactory, and Visual) and five action effectors (Foot/Leg, Hand/Arm, Head, Mouth, and Torso).

### Candidate LLMs and prompts

In the present study, we selected four LLMs based on a preliminary screening study. First, we compared the performance of the leading closed and open LLMs and identified the top-performing models from each category. From these, we chose two models of different sizes per category for our main experiments: GPT-4o-2024-05-13 (Gpt-4o) and GPT-4o-mini-2024-07-18 (Gpt-4o-mini) as closed models (OpenAI, 2024), and *Meta-Llama-3.1-405B-Instruct* (Llama-3.1-405B) and *Meta-Llama-3.1-70B-Instruct* (Llama-3.1-70B) as open models (Meta, 2024a). The inference parameters were fixed across all models (temperature = 0.60, top-p = .90). For Gpt-4o and Gpt-4o-mini, the seed value could not be deterministically fixed; therefore, a random seed was used.

All words were evaluated in 10 independent trials per psychological feature, and the average values were used as representative scores. For each psychological feature, we provided a system prompt specifying the role: “You are a native English speaker.” This was followed by a user prompt composed of three parts. The first part contained feature-specific instructions, formulated as closely as possible to those in the original articles. For example, for Babiness, the instruction read, “You will be presented with a list of words. For each word, please rate how much you associate it with babies on a scale between 1 (not associated with babies) to 10 (very associated with babies). Please use the full range of the scale and try to be as accurate as possible in your judgments.” If the original instructions included user interface-related phrases (e.g., “click the button”), these were removed and rephrased to be appropriate for LLM input (e.g., “rate the following word”). The second part of the user prompt concerned the response format: “Please respond in the format ‘word: number.’ Do not generate any text other than your response. If you are not familiar with a word’s meaning, please respond with the format ‘word: N’.” Because nonresponse was often permitted in human studies when participants were unfamiliar with a word, we included a similar instruction for the LLM. The final part was a list of the 695 target words. For each trial, all words were input simultaneously in a single concatenated prompt, and the model was instructed to output ratings for all words at once. This approach substantially reduced computational and financial costs compared to prompting each word individually. Importantly, the order of the words was randomly shuffled in each trial to minimize the risk of dilution effects (i.e., changes in rating accuracy or variability due to the fixed sequential presentation of words within a single prompt).

### Data analysis

We first examined the similarity of each lexical psychological feature between human participants and LLMs using Spearman’s rank correlation coefficient and Jensen–Shannon (JS) divergence. The former assesses whether words that receive higher or lower values from humans tend to receive similar higher or lower values from the LLMs. The latter quantifies the overall similarity between the two rating distributions by averaging their Kullback–Leibler (KL) divergences from their mean distribution. Unlike the KL divergence, the JS divergence is symmetric and remains finite even when the distributions have sparse tails or nonoverlapping supports, providing a stable measure of distributional difference. Smaller values (closer to 0) indicate greater similarity between the two distributions.

To capture the similarities and differences between human and LLM ratings in more detail, we also grouped the target words into four lexical categories based on the Wordbank classification (Braginsky et al., 2019; Frank et al., 2021): *Nouns* (316 items), *Predicates* (verbs and adjectives; 166 items), *Function words* (111 items), and *Other* (102 words). The category Other included sound effects (e.g., “moo,” “meow”), people (e.g., “boy,” “grandma”), and social routines (e.g., “hello,” “yes”). Analyzing LLM-derived psychological features within these categories allows us to examine whether the correspondence with human ratings varied across lexical classes.

We next assessed the degree to which the predictability of each psychological feature for AoA was similar or different between human participants and LLMs. We calculated Spearman's correlation coefficient between the words’ AoA and psychological features and the differences in these coefficients between humans and LLMs. Finally, we performed a data-driven cluster analysis to reveal which features could be promising for LLMs to be used as a replacement for human participants, and which features were not.

## Results

### Similarities of lexical psychological features between human and LLM ratings

#### Overall similarities across all words

**Fig. 1:**
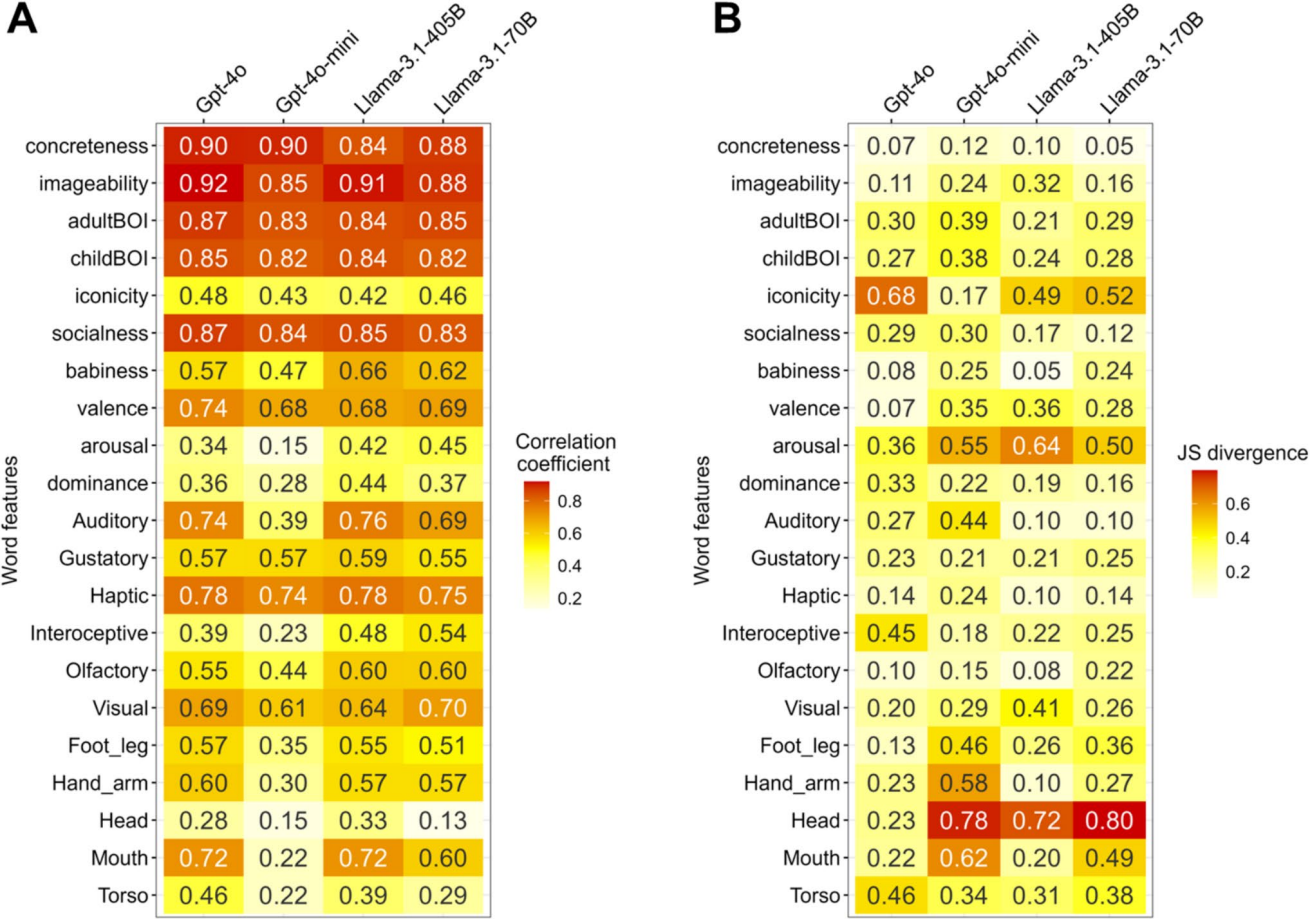
Similarity measures for each lexical psychological feature between human and LLM ratings. *Notes*. Spearman’s rank correlation coefficient (**A**) and Jensen–Shannon (JS) divergence (**B**). For correlations, higher values indicate higher similarities in terms of ranking between humans and LLMs. For the JS divergence, smaller values represent greater proximity between the two distributions

For some psychological features, such as Concreteness, Imageability, and BOI, all four candidate LLMs showed a strong positive correlation between human and model ratings (*r*s > .82; Fig. 1A). In contrast, other features, such as Iconicity, Arousal, and Head, estimated by LLMs did not correlate well with those estimated by human participants (e.g., for Arousal, *r*s < .45). When looking at similarities in terms of distribution, some features that demonstrated a high correlation between humans and LLMs often differed between the two sources, such that the distribution for Imageability did not resemble that of human ratings relatively well (JS divergence = .32 for Llama-3.1-405B; Fig. 1B).

**Fig. 2:**
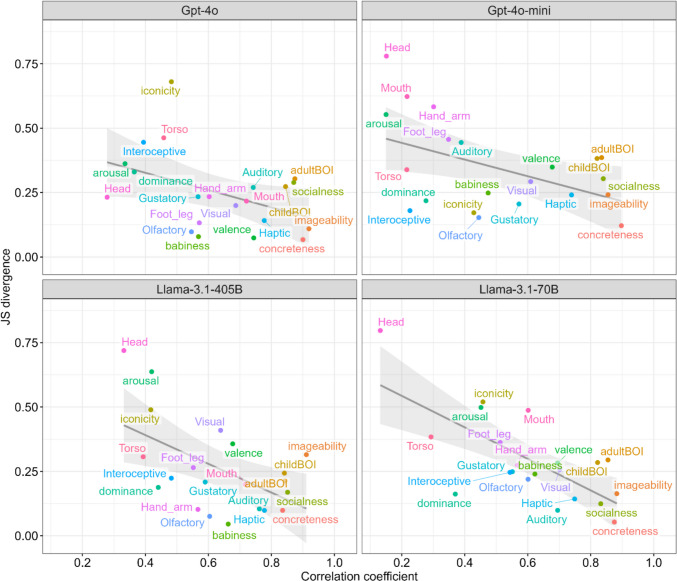
Relationships between rank correlation coefficients and JS divergences. *Notes*. The gray lines represent the regression lines, and the gray ribbons indicate 95% confidence intervals. In each panel, lexical psychological features located toward the lower right corner (e.g., Concreteness) indicate better alignment between the human and LLM ratings. Particularly for Llama-3.1-405B and Llama-3.1-70B, higher correlation coefficients were associated with a smaller JS divergence, suggesting that the two similarity metrics were well aligned

To make the relationship between the two metrics (i.e., rank correlation coefficient and JS divergence) immediately clear at a glance, we drew scatterplots, as shown in Fig. 2. We found significant negative correlations between the two metrics across all four models, indicating that features with higher correlation coefficients tended to have smaller JS divergence. This finding reflects good agreement between the two measures. Among them, Llama-3.1-405B (*r* = −.54, *p* = .010, 95% CI [−.79, −.15]) and Llama-3.1-70B (*r* = −.71, *p* < .001, 95% CI [−.87, −.40]) showed relatively stronger negative correlations, whereas the correlations were weaker for GPT-4o (*r* = −.44, *p* = .046, 95% CI [−.73, −.01]) and GPT-4o-mini (*r* = −.48, *p* = .027, 95% CI [−.76, −.06]).

**Fig. 3:**
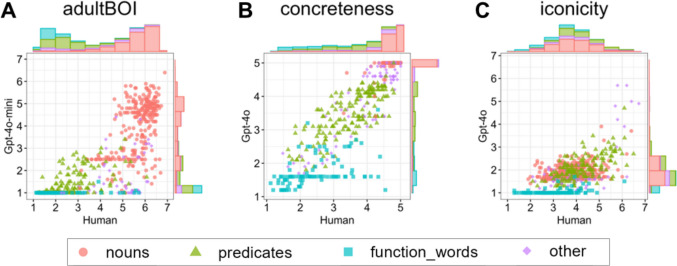
Examples of ratings for lexical psychological features between human participants and LLMs. *Notes*. Adult BOI rated using Gpt-4o-mini (**A**), and Concreteness (**B**) and Iconicity (**C**) rated using Gpt-4o. The figure shows scatter plots accompanied by marginal histograms. Colors indicate the lexical category (i.e., Nouns, Predicates, Function words, and Other). See the main text for descriptions of each example. An overview of all lexical psychological features across all the LLMs used is provided in the Supplementary Materials (Fig. S1)

Some features showed a discrepancy between their rank correlations and JS divergences. For example, in Gpt-4o-mini, Adult BOI showed a high correlation coefficient (*r*s = .83) but a relatively large JS divergence (JS divergence = .39). This suggests that while the rank ordering of words from low to high was preserved between the human and LLM ratings, the overall distributional shape differed substantially (see Fig. 3A). In other words, LLMs often reproduced the relative ordering of words but tended to compress or stretch the rating scale compared to human judgments. This distinction is important, particularly when downstream analyses rely on the absolute scale of ratings, such as when predicting AoA or behavioral responses.

#### Similarities by lexical category

It is interesting to note that even for psychological features demonstrating strong overall similarity, discrepancies still arose between human and LLM ratings depending on the lexical category. For example, when focusing on Concreteness, human ratings for Function words (e.g., prepositions and conjunctions) showed variability in perceived concreteness across words (e.g., “in” and “more” were perceived as more concrete than “of” or “because”), whereas the LLMs consistently rated these words as low in concreteness (Fig. 3B). A similar pattern was observed even for features with generally low similarity (e.g., Iconicity; Fig. 3C). While humans may form distinct psychological impressions not only for content words but also for function words, LLMs may treat function words as uniformly similar, potentially failing to capture nuanced differences.

**Fig. 4:**
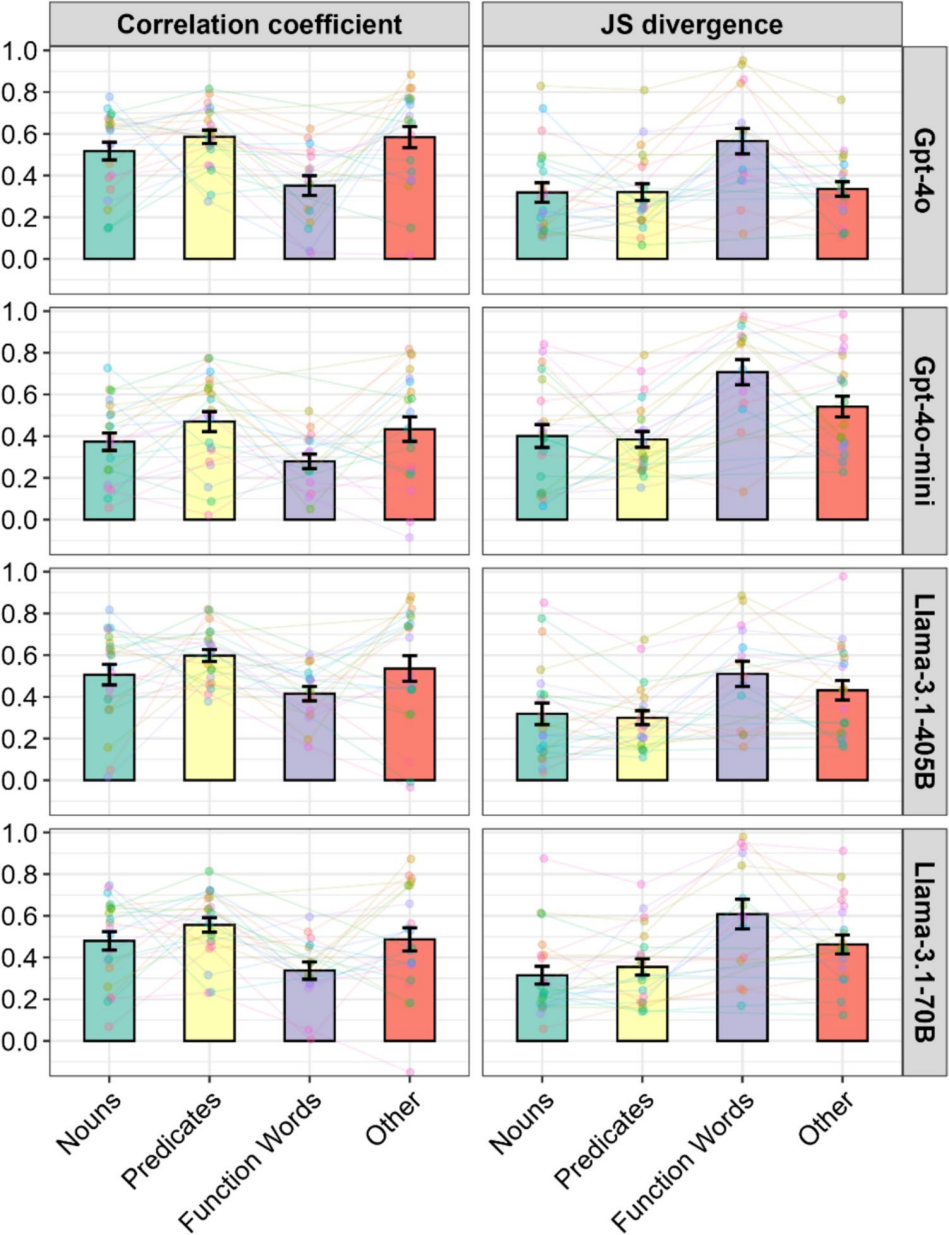
Mean similarity metrics by lexical category for each model. *Notes*. Bar plots represent the means and standard errors of the rank correlation coefficients and JS divergences. Individual data points (connected by lines) correspond to psychological features. Function words consistently exhibited the largest discrepancies between human and LLM ratings. See Fig. S2 in the Supplementary Materials for detailed visualizations of the distributions for each psychological feature

To move beyond anecdotal observations of individual features and to test whether the similarity between human and LLM ratings varied by lexical category, we collapsed the psychological features and computed the mean correlation coefficients and JS divergences (Fig. 4). We excluded Socialness, Valence, Arousal, and Dominance for Function words, as more than 90% of the human ratings were missing. In addition, Gustatory for Function words in GPT-4o, and both Gustatory and Olfactory for Function words in the other three models were excluded because the LLM ratings showed no variance, making the computation of correlations impossible (see Supplementary Table S2 for details on missing-value proportions).

We ran linear mixed-effects models with model (i.e., LLMs), lexical category, and their interaction as fixed effects, and psychological features as a random effect. For correlation coefficients, we found significant main effects of model (${\chi }^{2}$(3) = 28.98, *p* < .001) and category (${\chi }^{2}$(3) = 58.02, *p* < .001), but no significant interaction (${\chi }^{2}$(9) = 3.07, *p* = .96). Post hoc pairwise comparisons of lexical categories (Holm-corrected) revealed that Function words showed significantly weaker correlations than all other categories (against Nouns: *estimate* = 0.13, *SE* = 0.03, *p* < .001; Predicates: *estimate* = 0.21, *SE* = 0.03, *p* < .001; and Other: *estimate* = 0.17, *SE* = 0.03, *p* < .001). In addition, Nouns exhibited significantly weaker correlations than Predicates (*estimate* = 0.08, *SE* = 0.03, *p* = .0052). Regarding LLM comparisons, GPT-4o-mini showed the weakest correlations (compared with GPT-4o: *estimate* = 0.12, *SE* = 0.03, *p* < .001; Llama-3.1-405B: *estimate* = 0.12, *SE* = 0.03, *p* < .001; Llama-3.1-70B: *estimate* = 0.08, *SE* = 0.03, *p* = .025), whereas no significant differences were found among the remaining models (*p*s > .23).

For JS divergence, similar results were obtained: both model (${\chi }^{2}$(3) = 26.55, *p* < .001) and lexical category (${\chi }^{2}$(3) = 96.74, *p* < .001) showed significant main effects, while the interaction was not significant (${\chi }^{2}$(9) = 9.27, *p* = .41). Consistent with the correlation results, Function words showed larger JS divergences than all other categories, indicating greater distributional discrepancies between human and LLM ratings (against Nouns: *estimate* = 0.24, *SE* = 0.03, *p* < .001; Predicates: *estimate* = 0.24, *SE* = 0.03, *p* < .001; and Other: *estimate* = 0.14, *SE* = 0.03, *p* < .001). Other also exhibited significantly larger JS divergences than Nouns (*estimate* = 0.10, *SE* = 0.03, *p* < .001) and Predicates (*estimate* = 0.10, *SE* = 0.03, *p* < .001). Unlike the correlation coefficients, no significant difference between Nouns and Predicates was found (*estimate* = 0.00, *SE* = .03, *p* = .95). In LLM comparisons, again consistent with the correlation results, GPT-4o-mini showed the largest JS divergence (compared with GPT-4o: *estimate* = 0.12, *SE* = 0.03, *p* < .001; Llama-3.1-405B: *estimate* = 0.12, *SE* = 0.03, *p* < .001; Llama-3.1-70B: *estimate* = 0.07, *SE* = 0.03, *p* = .029), while the other models did not differ significantly (*p*s > .23).

In summary, Function words consistently showed the greatest divergence between human and LLM ratings across all models.

#### Predicting words’ age of acquisition (AoA) from psychological features: Human and LLM comparisons

Next, we examined the rank correlation coefficients between the AoA as an external measure and either human- or LLM-rated psychological features to evaluate the extent to which LLMs replicate human judgments from another perspective. As mentioned above, the AoA estimates were derived from Wordbank data (Frank et al., 2017) and served as a reference measure. Note that LLMs were not used for AoA estimations; they were used only to estimate the lexical psychological features of each word.

**Fig. 5:**
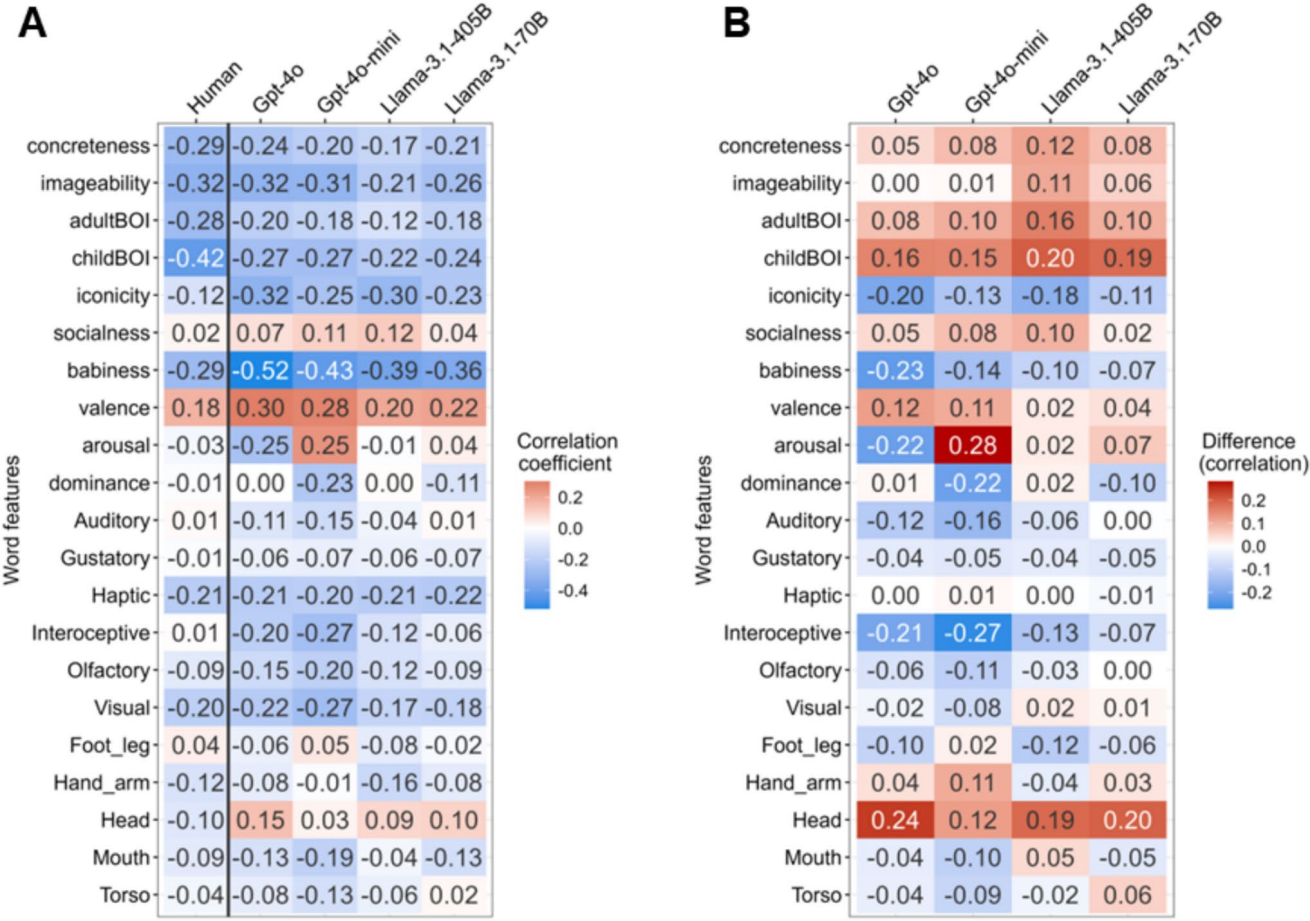
Predictability of AoA from each psychological feature rated by humans and LLMs. *Notes*. Spearman’s rank correlation coefficients between the word AoA and psychological features (**A**), and the differences in those correlations between humans and LLMs (**B**). In many cases, the correlations between AoA and psychological features rated by humans were similar to those between AoA and LLM-rated psychological features

Across all four LLMs tested, the correlations between AoA and psychological features rated by LLMs showed a certain similarity to those between AoA and human-rated psychological features (see Fig. 5). That is, features that exhibited higher correlations with AoA in human ratings tended to demonstrate similarly strong correlations in the LLM-based ratings. For instance, Concreteness rated by humans was negatively correlated with AoA (*r*s = −.29), suggesting that children tended to learn more concrete words earlier (Brysbaert et al., 2014; Tan et al., 2024). The same feature estimated by LLMs yielded comparable correlation values (e.g., Gpt-4o, *r*s = −.24; Llama-3.1-70B, *r*s = −.21). However, some discrepancies were also observed for certain features for which the predictive patterns differed between the human and LLM-based ratings. For instance, despite the fact that Arousal rated by humans did not correlate with AoA (*r*s = −.03), Gpt-4o produced a more substantial correlation (*r*s = −.25), and the features rated by Gpt-4o-mini differed not only in magnitude but also in the direction of correlations (*r*s = .25).

**Fig. 6:**
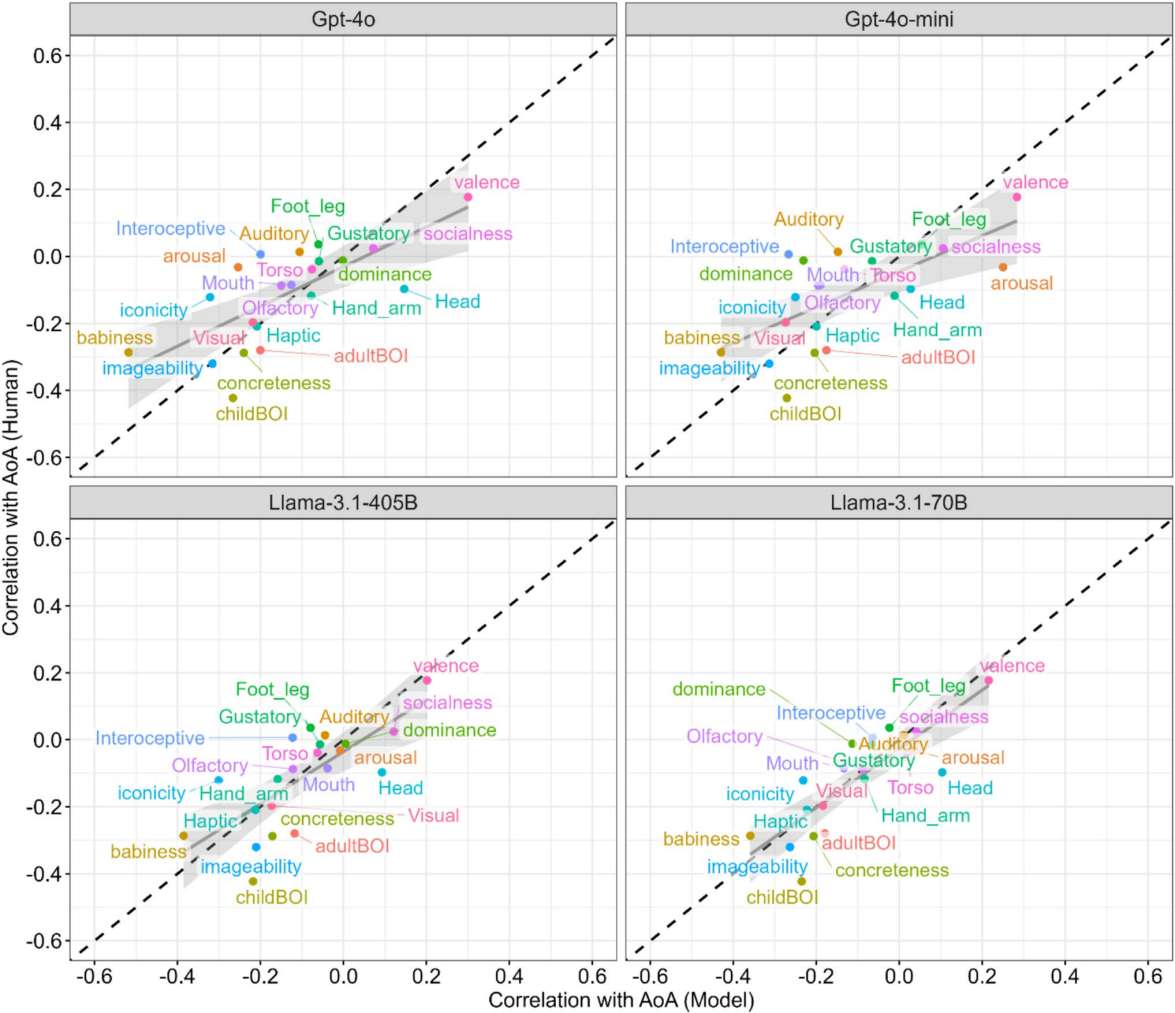
Relationship between rank correlation coefficients of AoA and psychological features rated by humans and LLMs. *Notes*. The gray lines represent the regression lines, and the gray ribbons indicate 95% confidence intervals. The closer the data points lie to the dashed diagonal line, the more similar the correlations with the AoA between human- and LLM-derived psychological feature ratings. For example, the regression line in Llama-3.1.70B closely aligns with the diagonal line, indicating a high similarity with human ratings. In contrast, the regression line for Gpt-4o is flatter than the diagonal line, suggesting that Gpt-4o tends to overestimate the predictability of AoA based on its own psychological feature ratings

To visualize these similarities and differences at a glance, Fig. 6 presents a scatter plot of the correlation coefficients between the AoA and each psychological feature rated by the two different sources. The closer the regression line lies to the dashed diagonal (i.e., *y* = *x*), the more similar the predictive patterns between humans and LLMs. Among the four LLMs, Llama-3.1-70B aligned with the diagonal most closely (*b* = 0.88, *r*s = .83), followed by Llama-3.1-405B (*b* = 0.78, *r*s = .73), Gpt-4o (*b* = 0.59, *r*s = .71), and Gpt-4o-mini (*b* = 0.53, *r*s = .66). The regression slopes for Gpt-4o and Gpt-4o-mini were flatter than the diagonal line, suggesting that these models tended to overestimate the strength of the correlation between psychological features and AoA. For instance, although the correlation between Babiness and AoA was −.29 in human rating, that in Gpt-4o rating was −.52. Similarly, the correlation between Valence and AoA was .18 in human rating, and that in Gpt-4o rating was .30.

To test whether the regression line significantly deviated from the identity line (i.e., slope, *b* = 1), we fitted linear models predicting human–AoA correlations from model–AoA correlations for each LLM. The regression slopes were significantly smaller than 1 (i.e., flatter regression lines) for Gpt-4o (*F*(1, 19) = 9.26, *p* = .0066, 95% CI [0.31, 0.87]) and Gpt-4o-mini (*F*(1, 19) = 11.62, *p* = .0029, 95% CI [0.25, 0.82]), confirming their tendency to overestimate AoA associations relative to human ratings. The regression slopes did not differ significantly from 1 for the other two models (Llama-3.1-405B: *F*(1, 19) = 1.78, *p* = .19, 95% CI [0.43, 1.13]; Llama-3.1-70B: *F*(1, 19) = 0.80, *p* = .38, 95% CI [0.59, 1.16]).

To further ensure that the observed patterns were not merely driven by global lexical factors, we conducted supplementary analyses controlling for Concreteness and word frequency, variables known to be strongly associated with AoA (e.g., Brysbaert et al., 2014; Hills et al., 2009). For this purpose, human-derived Concreteness ratings and word frequency in child-directed speech were included as control variables, and partial correlations between AoA and each psychological feature were calculated. The frequency measures were computed from the CHILDES corpus (MacWhinney, 2000) following the procedures described by Sánchez et al. (2019). The overall pattern of results remained qualitatively consistent with the main analyses (see Figs. S3 and S4). For example, as shown in Fig. 5B, Child BOI generally showed relatively large discrepancies between human- and model-derived correlations with AoA across all models. Moreover, both GPT-4o and GPT-4o-mini continued to show a tendency to overestimate the predictability of AoA from psychological features even after controlling for the two global factors (*p*s < .010).

### Clustering psychological features by human-LLM similarities

**Fig. 7:**
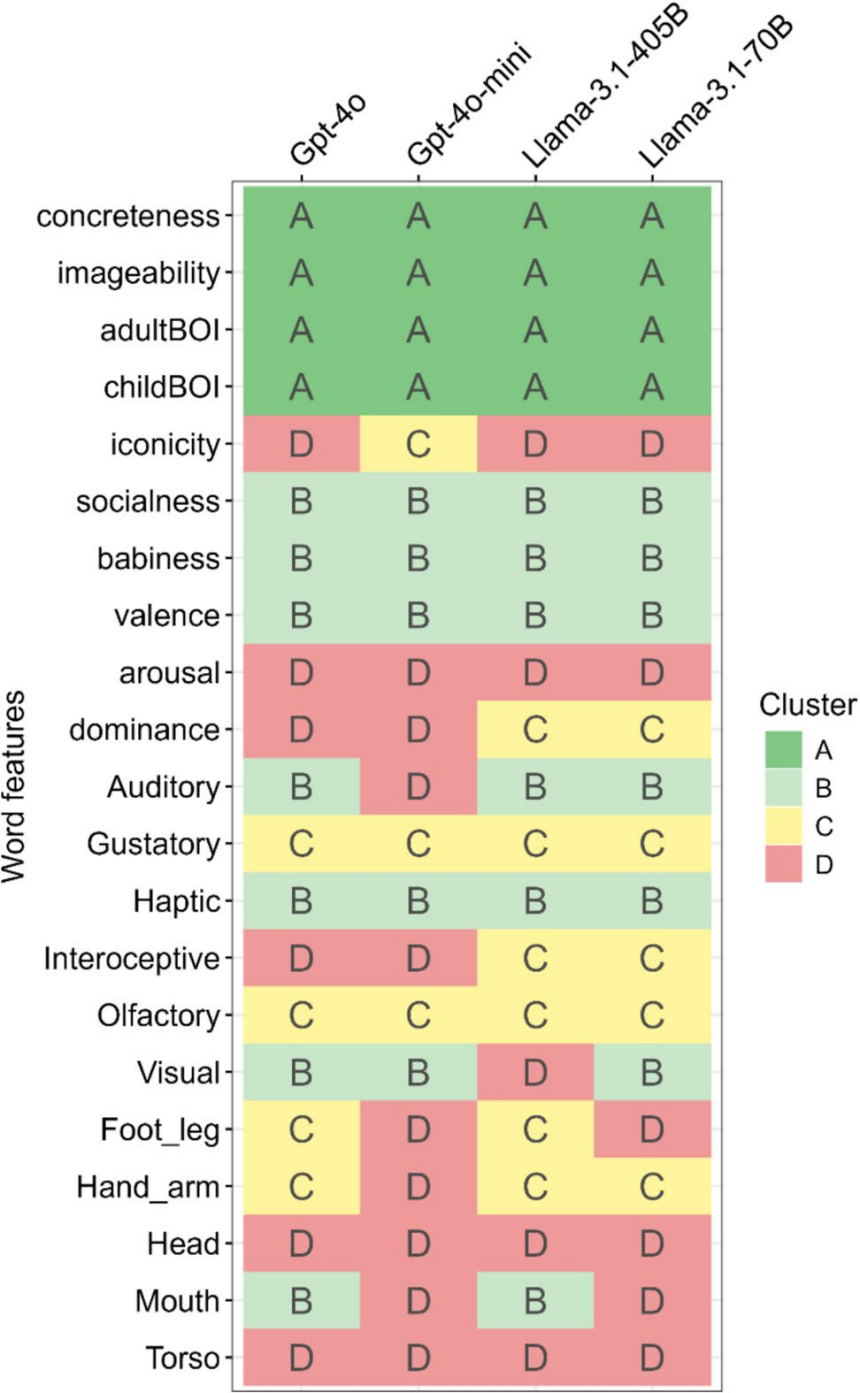
Result of clustering psychological features in each model based on the human–model similarities. *Notes*. The *k*-means approach was applied to clustering, following t-SNE dimensionality reduction. Clusters A and B represent features with higher human–model similarity. For interpretation of the cluster characteristics, see Fig. 8

Our last analysis concerns for which psychological features LLMs can potentially replace human participants and which features they cannot. To facilitate the clustering of psychological features, we first applied t-SNE (van der Maaten & Hinton, 2008), which is a nonlinear dimension-reduction technique, to the four summary measures that reflect the human-LLM similarities mentioned above: correlations between human and LLM ratings, JS divergence between human- and LLM-rating distributions, correlations with AoA, and differences in correlations with AoA between humans and LLM ratings (Fig. S5). Based on the resulting two-dimensional space, we performed *k*-means clustering and identified four distinct clusters (A–D). The features assigned to each cluster were largely consistent across the LLMs, although some variation was observed (Fig. 7). Concreteness, Imageability, and both Adult and Child BOI were consistently grouped into Cluster A across all models, whereas Iconicity, Arousal, Head, and Torso were assigned to Cluster D in most models.

**Fig. 8:**
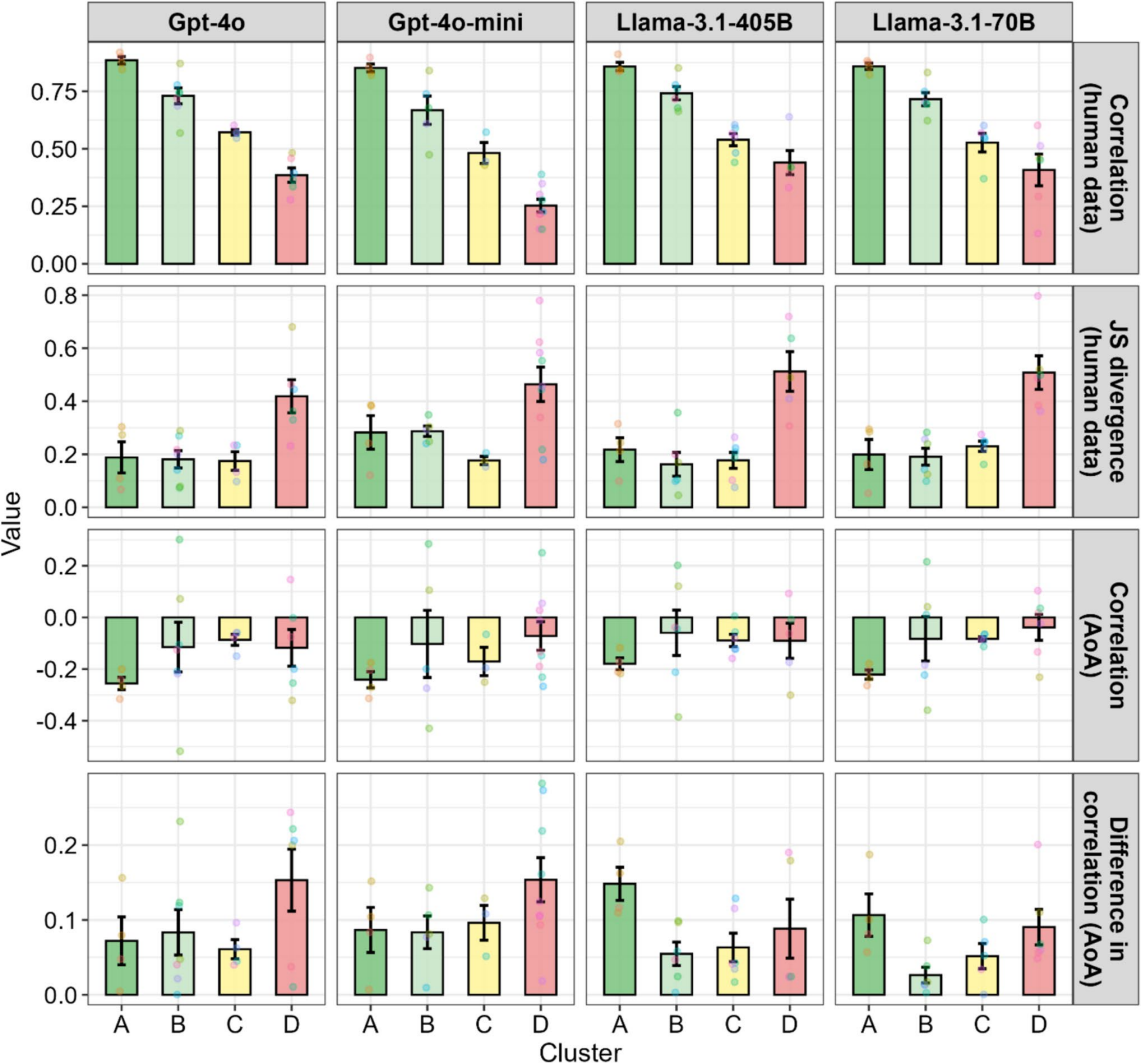
Mean similarity indices between human and model ratings across clusters. *Notes*. Clusters A and B generally exhibited higher similarities to human ratings, characterized by stronger correlations and smaller JS divergences. In contrast, similarities decreased toward Clusters C and D, with Cluster D showing particularly large JS divergences and greater discrepancies in correlations with AoA

To interpret the characteristics of each cluster, we visualized how the four identified clusters differed for each model in terms of the original similarity measures (Fig. 8). Across all models, similarities with human ratings generally decreased from Cluster A to Cluster D. Compared to Clusters A and B, Clusters C and D tended to show smaller correlation coefficients and larger JS divergences, with Cluster D exhibiting particularly large distributional discrepancies. Regarding the differences in correlation with AoA based on human ratings, Cluster D showed the greatest divergence overall, although some deviations were also occasionally observed for Cluster A.

Overall, features belonging to Clusters A and B (e.g., Concreteness, Imageability, Adult BOI, and Child BOI) may have the potential to serve as human substitutes. However, as noted above, features in Cluster A often tended to overestimate the predictability of AoA, even in the Llama models, where no overestimation was observed when aggregating all features. Thus, when balancing predictive accuracy and stability, the features grouped in Cluster B (e.g., Socialness, Babiness, Valence, and Haptic) may represent more modest yet reliable candidates depending on the purpose of use. By contrast, features that were consistently assigned to Cluster D in at least two models (i.e., Iconicity, Arousal, Dominance, Interoceptive, Foot/Leg, Head, Mouth, and Torso) appeared less reflective of human intuitive cognition. Model-specific malfunctions were also observed. For example, Auditory in GPT-4o-mini and Visual in Llama-3.1-405B were far from similar to human ratings, whereas these features showed relatively higher similarities in the other LLMs.

We note that, to confirm that the observed clustering patterns were not artifacts of the t-SNE embedding, we also performed an analysis using uniform manifold approximation and projection (UMAP; McInnes et al., 2018) followed by Gaussian mixture modeling (GMM), instead of the t-SNE and *k*-means pipeline. The UMAP–GMM solution yielded a qualitatively similar organization of features across all models (see Supplementary Figs. S6 and S7), consistent with the t-SNE-based results. The adjusted Rand index (ARI) between the two clustering solutions was .69, indicating reasonable agreement.

## Discussion

In this study, we used four state-of-the-art LLMs to evaluate 21 static psychological features of 695 English words acquired early by young children. Throughout the analysis, we examine the similarities and differences between LLM and human ratings from various perspectives to discuss the applicability of LLMs in cognitive science. The results revealed that several features, such as Concreteness or Adult and Child BOI, demonstrated a high resemblance between the two sources. As Concreteness is a widely used measure in cognitive science and psychology (Kewenig et al., 2025), the finding that LLMs can approximate human ratings suggests a promising practical replacement for human data. Recent studies have begun to explore transformer-based approaches to automatically predict Concreteness values (e.g., Kewenig et al., 2025), offering broad lexical coverage and eliminating concerns regarding missing values. In our study, several psychological features had substantial missing human rating data, whereas the LLM-based ratings were nearly complete across all target words (Supplementary Table S2).

This methodological advantage was complemented by theoretical insights. Some researchers have hypothesized that LLMs, which do not directly interact with the world, contain embedded world knowledge because humans implicitly encode information about the world into language (Taniguchi et al., 2024) and/or because the distributional structure of language reflects world knowledge (Kauf et al., 2023). Supporting this view, Gurnee and Tegmark (2023) showed that LLMs can capture the spatial and temporal structures of the world. Our findings provide further evidence that linguistic distribution, at least for some psychological features, carries structured knowledge grounded in human cognition.

While the LLMs showed moderate to high alignment with human judgments for some psychological features, our results also revealed clear discrepancies for other features, such as Iconicity and Arousal. These discrepancies may stem from the fundamental limitations of LLMs, including their lack of multimodal grounding, embodiment, and nuanced understanding of figurative language (e.g., Barrett & Stout, 2024; Bisk et al., 2020; Wicke, 2023). Regarding the Iconicity rating, human participants assessed the similarity between a word’s sound and its meaning after pronouncing it aloud (Winter et al., 2023). In contrast, LLMs that do not possess articulatory experience or sensorimotor systems cannot simulate or evaluate phonological–motor mappings. Another type of divergence was observed in function words such as prepositions. While humans can perceive certain psychological properties, even for such non-content words, LLMs show difficulty in doing so. This may also be due to their lack of embodiment.

It should also be noted that the present study focused on static lexical judgments, where each word was evaluated in isolation. In contrast, human language understanding is inherently dynamic, continuously updating representations as contextual information accumulates (Baldassano et al., 2017; Zadbood et al., 2022). Future research could explore whether incorporating context-dependent information allows LLMs to approximate human-like judgments more closely. For instance, providing temporal and contextual cues more explicitly may improve model–human alignment for certain features (e.g., Iconicity and Arousal). Although obtaining such context-sensitive ratings from human participants would be time-consuming and costly, LLMs may offer a practical way to simulate dynamic, contextually grounded psychological evaluations at scale.

This study makes three main contributions to the literature. First, we identified the strengths and limitations of LLMs in rating lexical psychological features. By evaluating how well human psychological properties are embedded in LLMs, this work contributes to a deeper understanding of “machine psychology” (Binz & Schulz, 2023; Frank & Goodman, 2025; Hagendorff et al., 2024) and aligns with recent proposals that frame LLMs as psychological simulators within cognitive science (Lin, 2025). The findings provide useful insights for future model development, such that the poor performance on Iconicity suggests that incorporating auditory information (e.g., sound spectrograms) may bring models closer to human-like representations. Second, we examined how and to what extent LLMs can be used to advance research on language development and psycholinguistics. Even in English, where training data are considered abundant, LLMs’ performance was not yet sufficient, and some models demonstrated biases in terms of predicting the words’ AoA.

Nevertheless, LLMs may still be useful for preliminary investigations, particularly when developing new psychological features, extending analyses to less-resourced languages, or filling in missing values. Recent studies on automated cross-linguistic Concreteness ratings support this potential (Kewenig et al., 2025). Third, this study provides a new framework for evaluating LLMs’ cognitive abilities that complements the existing benchmarks. Rather than focusing on complex tasks (e.g., Cho et al., 2025; Hendrycks et al., 2021; Liang et al., 2023; Meta, 2024b), we assessed the model responses to early-acquired, relatively simple words, revealing meaningful discrepancies with human data. The measures and procedures used in this study would serve as new benchmarks for evaluating the proximity of LLMs’ cognition to humans.

Our study also complements and extends recent work by Trott (2024), who similarly investigated the extent to which Gpt-4 reflects human psychological ratings across a range of lexical norms. While both studies share the goal of evaluating the cognitive plausibility of LLM-derived features, our work contributes additional methodological and developmental perspectives. Specifically, we compared multiple LLMs, including Gpt-4o and open-source models, allowing for broader insight into model-specific variability. Moreover, our analysis focused on early-acquired words relevant to language development, with lexical items carefully selected based on developmental criteria. We also examined variation across parts of speech (e.g., nouns and predicates), which has not been explicitly addressed in prior work. These extensions provide a more nuanced understanding of how LLMs align with human psychological representations in the context of early word learning.

This study has several limitations that open avenues for future research. One possibility is that LLM-based estimates of psychological features could be better approximated to those of humans by calibrating the models using a subset of human ratings. As a follow-up study, we explored this idea by providing 10–50% of the human-rated features for the target words as calibration data and evaluated the resulting outputs for the remaining words (see Supplementary Fig. S8). The calibration and test sets were balanced across lexical categories (e.g., Nouns and Predicates) to ensure comparability. However, this hybrid procedure yielded no clear improvement in model performance, suggesting that simple partial calibration may be insufficient and that more sophisticated hybrid human–LLM strategies are needed.

Another promising direction is to develop prompting strategies that enable LLMs to reproduce not only mean ratings but also the variability observed in human judgments. In the present study, the variance of LLM-generated ratings across 10 trials for each word was smaller than that reported in human norms (see Fig. S9). While LLMs can approximate average human ratings to some extent, they still fall short in capturing the full distributional variability of individual judgments, which may partly result from the use of a single, fixed role in our prompts. Introducing more diverse contextual roles (e.g., specifying age, gender, or background), or prompting the model to generate a distribution of possible ratings rather than a single value, can help simulate the diversity inherent in human responses. This approach aligns with recent efforts to approximate the distributional patterns of human behavior using LLMs (Binz et al., 2025). In fact, the finding that Gpt-4o and Gpt-4o-mini showed stronger predictability of AoA than human ratings may reflect not model overestimation, but rather greater noise in the human norms themselves. Additionally, midscale human ratings (i.e., those near the midpoint of the scale) are often associated with judgmental disagreement among participants (Pollock, 2018). Given that the LLMs exhibited relatively low variability across trials, such disagreement effects are likely minimal in this study. However, future work could explore how varying role assignments might induce disagreement-like variability, potentially offering insights into the cognitive mechanisms that give rise to individual differences in human judgment.

Furthermore, differences in performance across LLMs raise a central question of *why* some models approximate human psychological ratings more closely than others. Closed commercial models such as Gpt-4o often achieve superior performance to open models in cognitive and psycholinguistic tasks (Strachan et al., 2024), yet their internal mechanisms remain opaque because only input–output behavior is observable. In contrast, open-weight models such as Meta-Llama provide full accessibility and reproducibility, allowing researchers to analyze internal representations and experimentally manipulate model size or training data where available. As Binz et al. (2025) demonstrated, large open-weight models can be systematically adapted for cognitive research, allowing their internal representations to be examined and aligned with human behavioral and neural data. Thus, closed models can serve as a practical upper bound or reference point for human–LLM correspondence, whereas open models allow researchers to gain mechanistic insight and theoretical progress (see also Section 4 in Conde et al., 2025, for a guide for researchers). This complementary relationship underscores the importance of jointly studying both types of models. Moreover, fully open initiatives such as OLMo 2 (OLMo et al., 2024), which publicly release not only training data and model weights but also thousands of intermediate checkpoints, enable a new kind of *developmental* or *ontogenic* investigation that traces how human-like representations emerge as training progresses. Integrating these two lines of research will advance both the methodological rigor and the theoretical depth of cognitive modeling with LLMs, helping to clarify how such models internalize psychological and linguistic structure while ensuring transparency and reproducibility.

This study offers methodological insights into the evaluation of LLMs as approximators of human psychological representations. By systematically comparing the LLM-derived ratings with human norms across multiple psychological features, we provide a practical framework for assessing the cognitive plausibility of LLMs. Our findings demonstrate that although LLMs can replicate certain patterns in human judgments, notable divergences remain. Our approach probes model performance and model–human alignment in interpretable and testable ways.

## Supplementary Information

Below is the link to the electronic supplementary material.

Supplementary Material 1 (PDF 3.87 MB)

## Data Availability

The data used in the present study can be found at https://github.com/hagi-hara/psychfeaturesllm.

## Code availability

Our prompts, codes and exported ratings estimated by LLMs can be found and are available at https://github.com/hagi-hara/psychfeaturesllm.
