# Supplementary material for: How well do large language models mirror human cognition of word concepts?: A comparison of psychological ratings for early-acquired English words
Source: Behav Res Methods. 2026 Feb 2;58(2):58. doi: 10.3758/s13428-025-02938-2 (PMC12864368; doi:10.3758/s13428-025-02938-2)
Supplement: Supplementary file 1 — Supplementary Material 1 (PDF 3.87 MB) [file 13428_2025_2938_MOESM1_ESM.pdf]

Supplementary Materials

Table S1

Numbers and proportions of atypical word items in the 695 target words

Category	# Items	Multiple word	Plural forms	Polysemous/ homophonous
Nouns	316	08 (2.5%)	13 (4.1%)	23 (7.3%)
Predicates	166	00 (0.0%)	00 (0.0%)	16 (9.6%)
Function words	111	18 (16.2%)	00 (0.0%)	02 (1.8%)
Other	102	03 (2.9%)	00 (0.0%)	02 (2.0%)
Total	695	29 (4.2%)	13 (1.9%)	43 (6.2%)

Notes. Atypical word items include (a) *multiple-word items* combining synonyms or variants (e.g., "soda/pop", "tissue/kleenex"), (b) *plural forms* (e.g., "bubbles", "carrots"), and (c) *polysemous or homophonous items* with meanings specified in parentheses (e.g., "chicken (animal/food)", "watch (object/action)"). As for (c), some items included a specified meaning while their alternative meanings were not listed (e.g., "rock (object)" was included, whereas "rock (action)" was not). Across categories, such atypical words accounted for approximately 2–6% of the total, suggesting that they have minimal influence on the overall results. Word categories follow the Wordbank classification (Braginsky, 2024; Frank et al., 2017) and are grouped into *nouns*, *predicates* (verbs and adjectives), *function words*, and *other* (e.g., sounds such as "moo", "meow", social routines such as "hello", "yes", or people such as "boy", "grandma").

Table S2

Numbers and proportions of words with missing ratings

Word features	Human ratings (# word items)				
	Nouns (316)	Predicates (166)	Function words (111)	Other (102)	Total (695)
Concreteness	8 (2.5%)	1 (0.6%)	17 (15.3%)	21 (20.6%)	47 (6.8%)
Imageability	171 (54.1%)	37 (22.3%)	40 (36.0%)	68 (66.7%)	316 (45.5%)
Adult BOI	30 (9.5%)	3 (1.8%)	20 (18.0%)	28 (27.5%)	81 (11.7%)
Child BOI	19 (6.0%)	1 (0.6%)	13 (11.7%)	28 (27.5%)	61 (8.8%)
Iconicity	24 (7.6%)	2 (1.2%)	16 (14.4%)	24 (23.5%)	66 (9.5%)
Socialness	228 (72.2%)	97 (58.4%)	108 (97.3%)	76 (74.5%)	509 (73.2%)
Babiness	65 (20.6%)	12 (7.2%)	18 (16.2%)	32 (31.4%)	127 (18.3%)
Valence	27 (8.5%)	7 (4.2%)	102 (91.9%)	44 (43.1%)	180 (25.9%)
Arousal	27 (8.5%)	7 (4.2%)	102 (91.9%)	44 (43.1%)	180 (25.9%)
Dominance	27 (8.5%)	7 (4.2%)	102 (91.9%)	44 (43.1%)	180 (25.9%)
Auditory	10 (3.2%)	1 (0.6%)	17 (15.3%)	21 (20.6%)	49 (7.1%)
Gustatory	10 (3.2%)	1 (0.6%)	17 (15.3%)	21 (20.6%)	49 (7.1%)
Haptic	10 (3.2%)	1 (0.6%)	17 (15.3%)	21 (20.6%)	49 (7.1%)
Interoceptive	10 (3.2%)	1 (0.6%)	17 (15.3%)	21 (20.6%)	49 (7.1%)
Olfactory	10 (3.2%)	1 (0.6%)	17 (15.3%)	21 (20.6%)	49 (7.1%)
Visual	10 (3.2%)	1 (0.6%)	17 (15.3%)	21 (20.6%)	49 (7.1%)
Foot/Leg	10 (3.2%)	1 (0.6%)	17 (15.3%)	21 (20.6%)	49 (7.1%)
Hand/Arm	10 (3.2%)	1 (0.6%)	17 (15.3%)	21 (20.6%)	49 (7.1%)
Head	10 (3.2%)	1 (0.6%)	17 (15.3%)	21 (20.6%)	49 (7.1%)
Mouth	10 (3.2%)	1 (0.6%)	17 (15.3%)	21 (20.6%)	49 (7.1%)
Torso	10 (3.2%)	1 (0.6%)	17 (15.3%)	21 (20.6%)	49 (7.1%)

Notes. The numbers and proportions of words with missing ratings in the human norms are shown. Darker shading (from white to deep red) indicates a higher proportion of missing values. For the human ratings, some psychological features had substantial missing values among the 695 target words. In contrast, the LLM-based ratings were almost complete. In this study, the LLM ratings for each psychological feature were the average of ten independent trials. Therefore, words marked

as missing indicate cases where the LLMs either skipped the response or failed to provide an answer in the correct format in all ten trials. Among all the LLMs used, only three words were completely missing, all from the Imageability ratings in Llama-3.1-405B. These three words were related to proper nouns (“babysitter’s name”, “child’s own name”, and “pet’s name”), and the model consistently output “N” across all ten trials, indicating that it did not recognize the meaning of the expressions. This likely occurred because the model treated them as referring to unspecified personal names rather than meaningful lexical concepts. Note that these three words were not included in the human norms for any of the psychological features, and therefore did not affect the results.

Figure S1

Ratings for lexical psychological features between human participants and LLMs

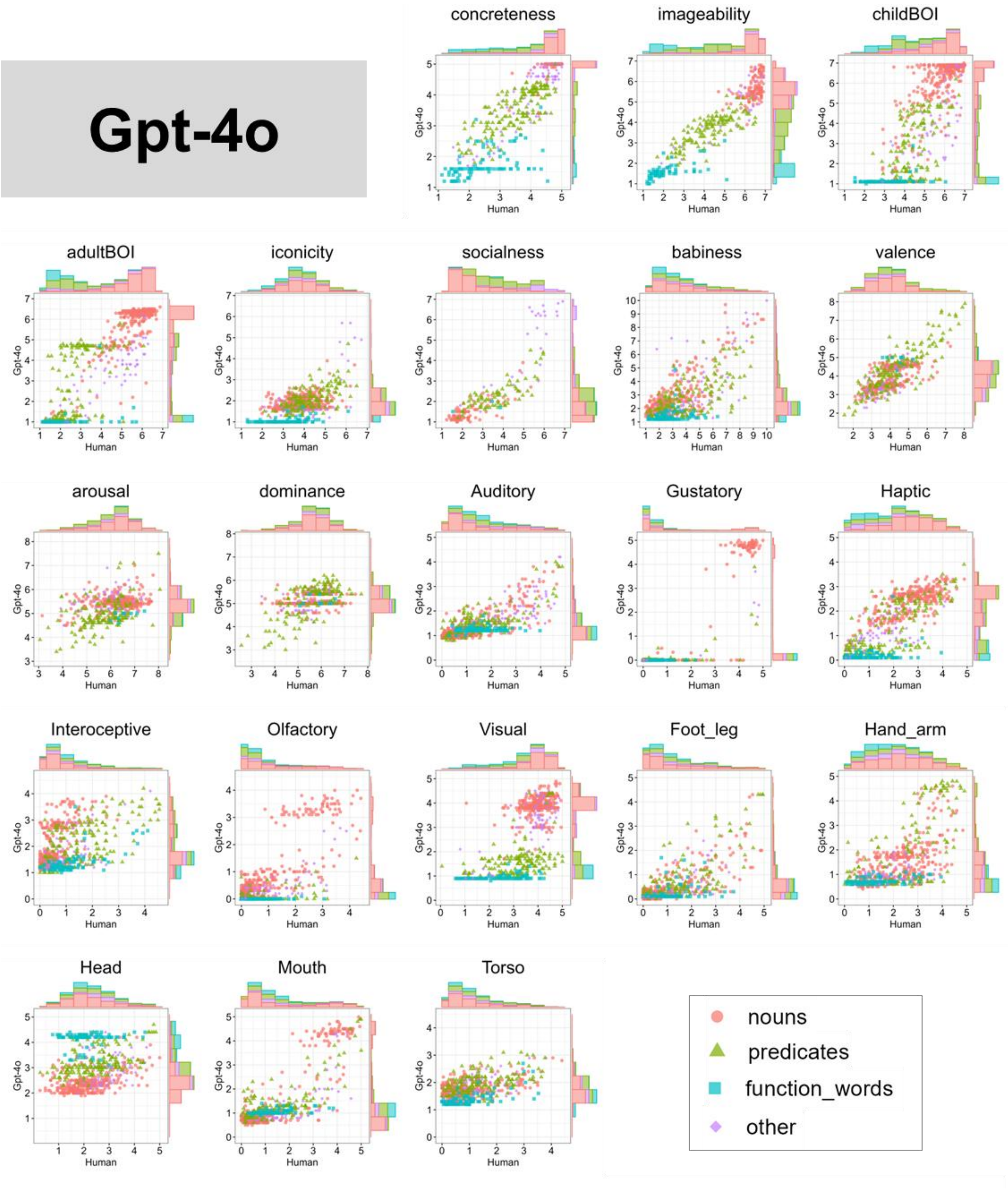

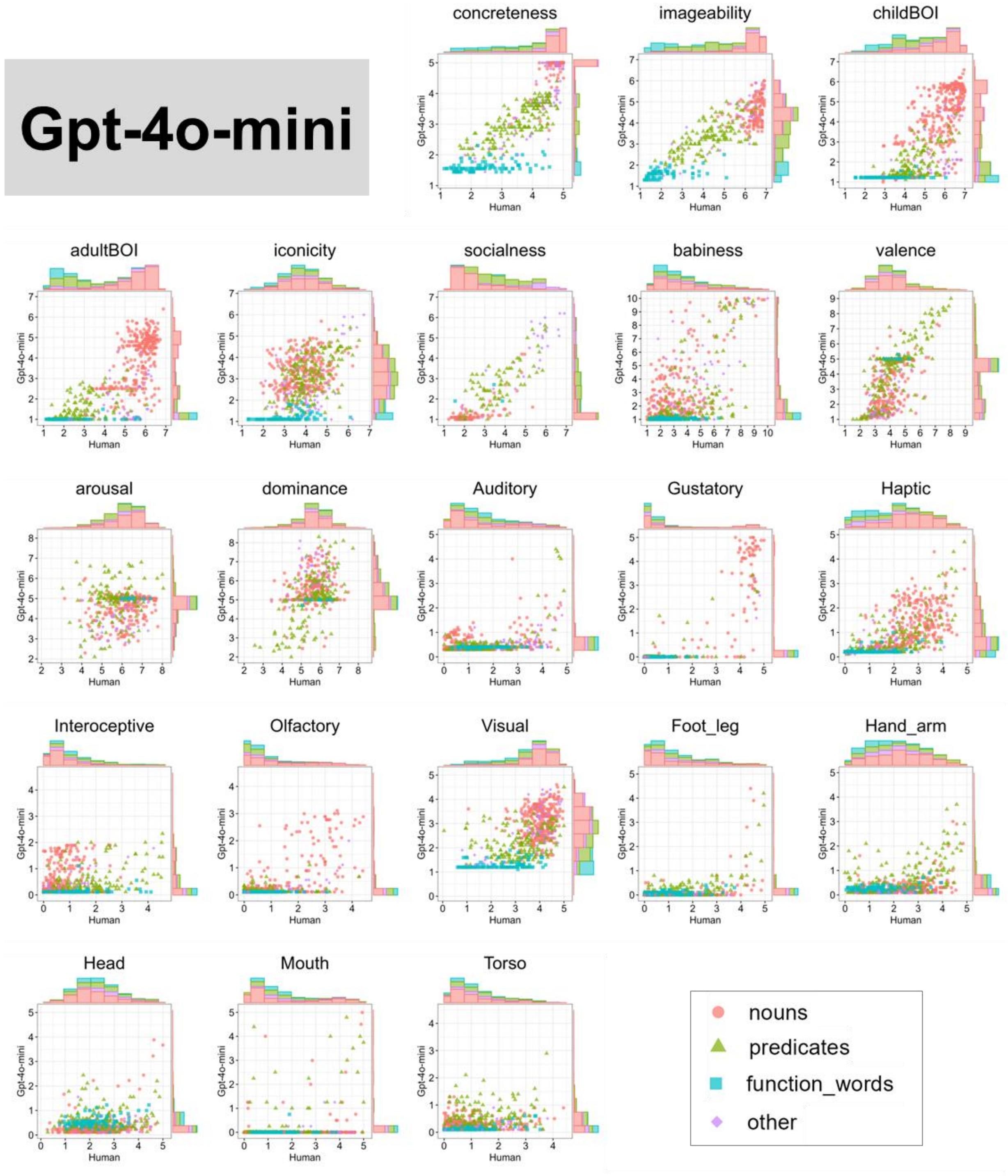

Gpt-4o-mini
concreteness
imageability
childBOI
adultBOI
iconicity
socialness
babiness
valence
arousal
dominance
Auditory
Gustatory
Haptic
Interoceptive
Olfactory
Visual
Foot_leg
Hand_arm
Head
Mouth
Torso
Human
Gpt-4o-mini
nouns
predicates
function_words
other

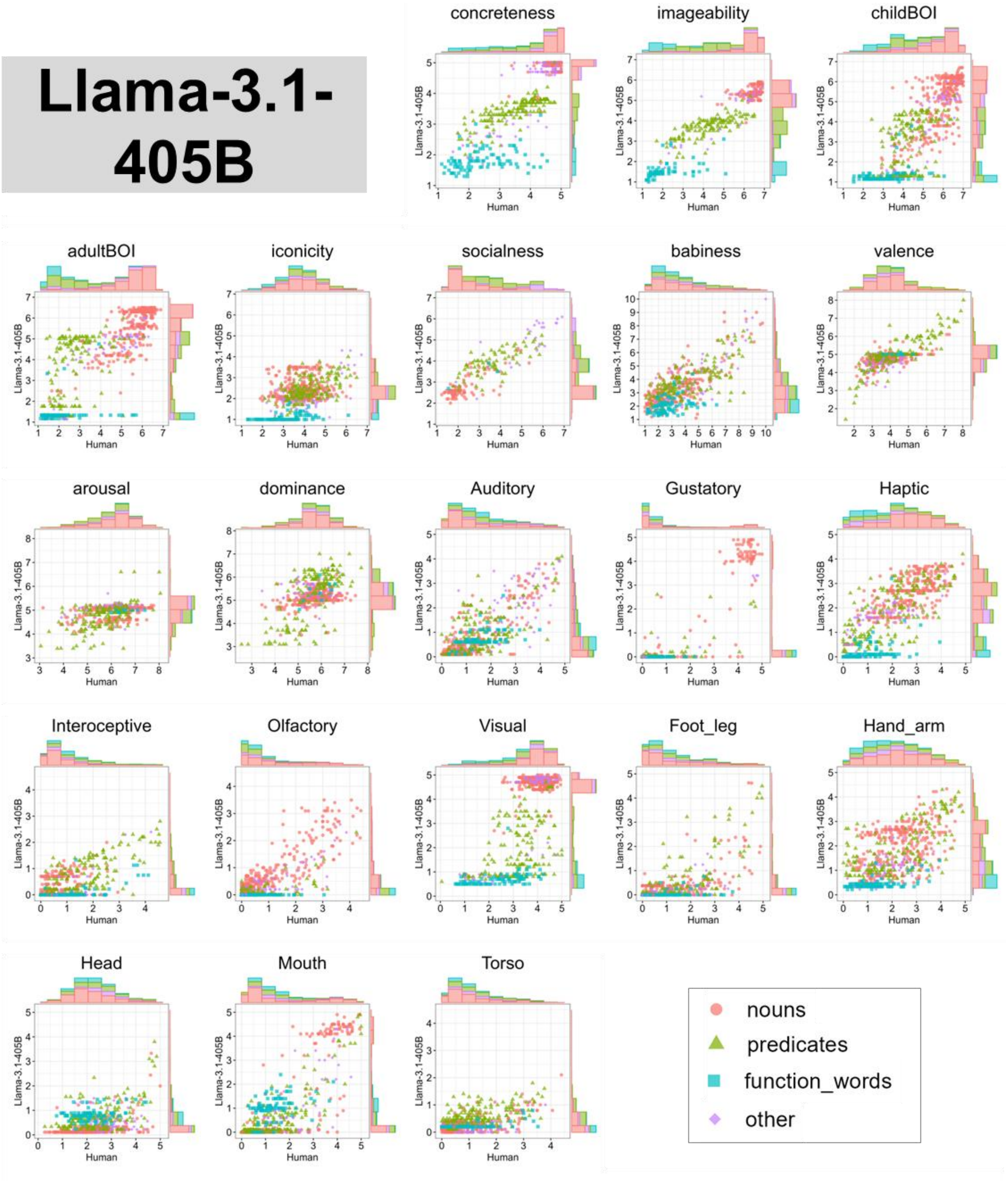
Llama-3.1-405B
concreteness
imageability
childBOI
adultBOI
iconicity
socialness
babiness
valence
arousal
dominance
Auditory
Gustatory
Haptic
Interoceptive
Olfactory
Visual
Foot_leg
Hand_arm
Head
Mouth
Torso
Human
Llama-3.1-405B
nouns
predicates
function_words
other

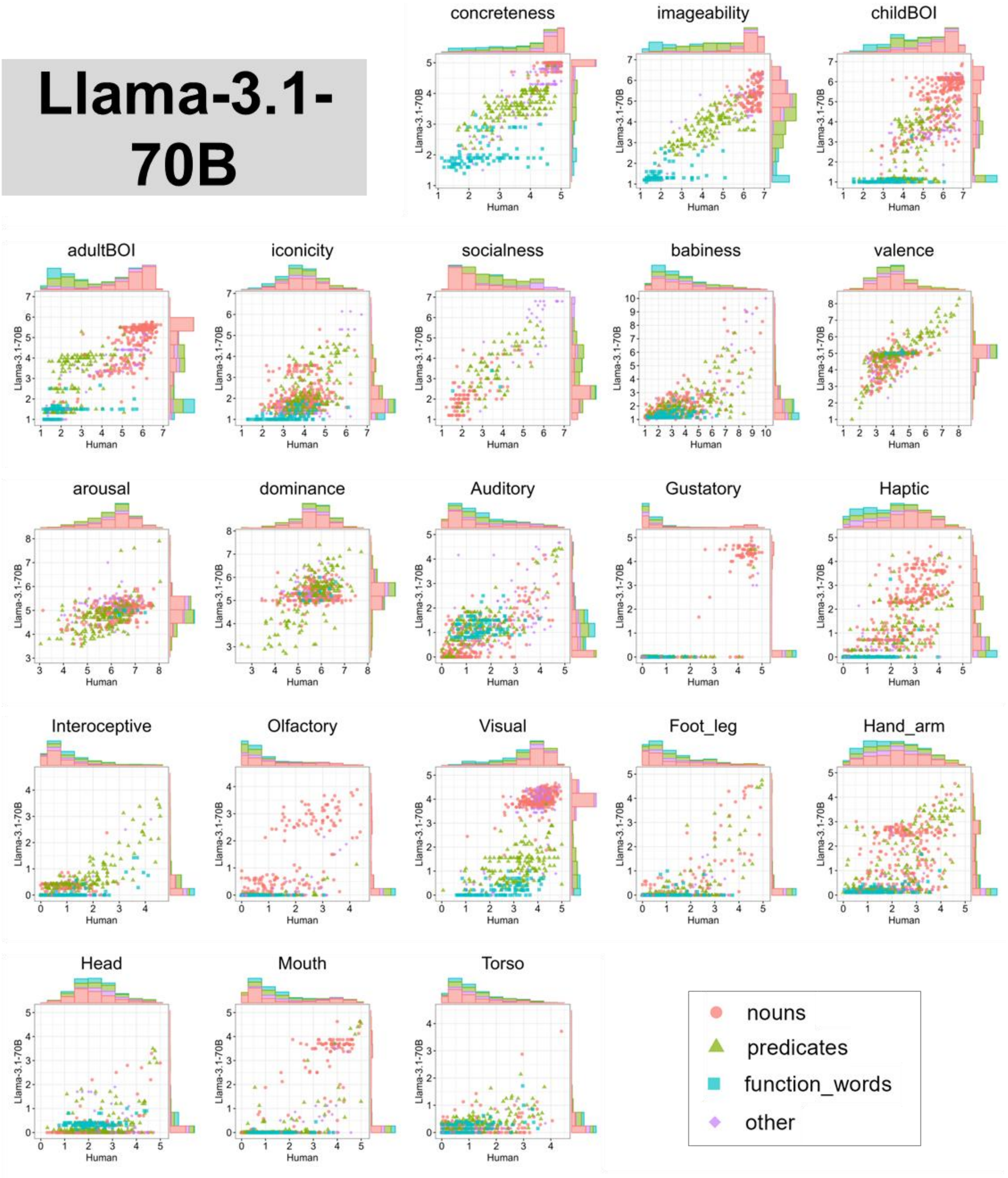

Notes. The figure shows scatter plots accompanied by marginal histograms. Colors indicate the lexical category (i.e., Nouns, Predicates, Function words, and Other).

Figure S2

Relationships between rank correlation coefficients and JS divergences across lexical categories

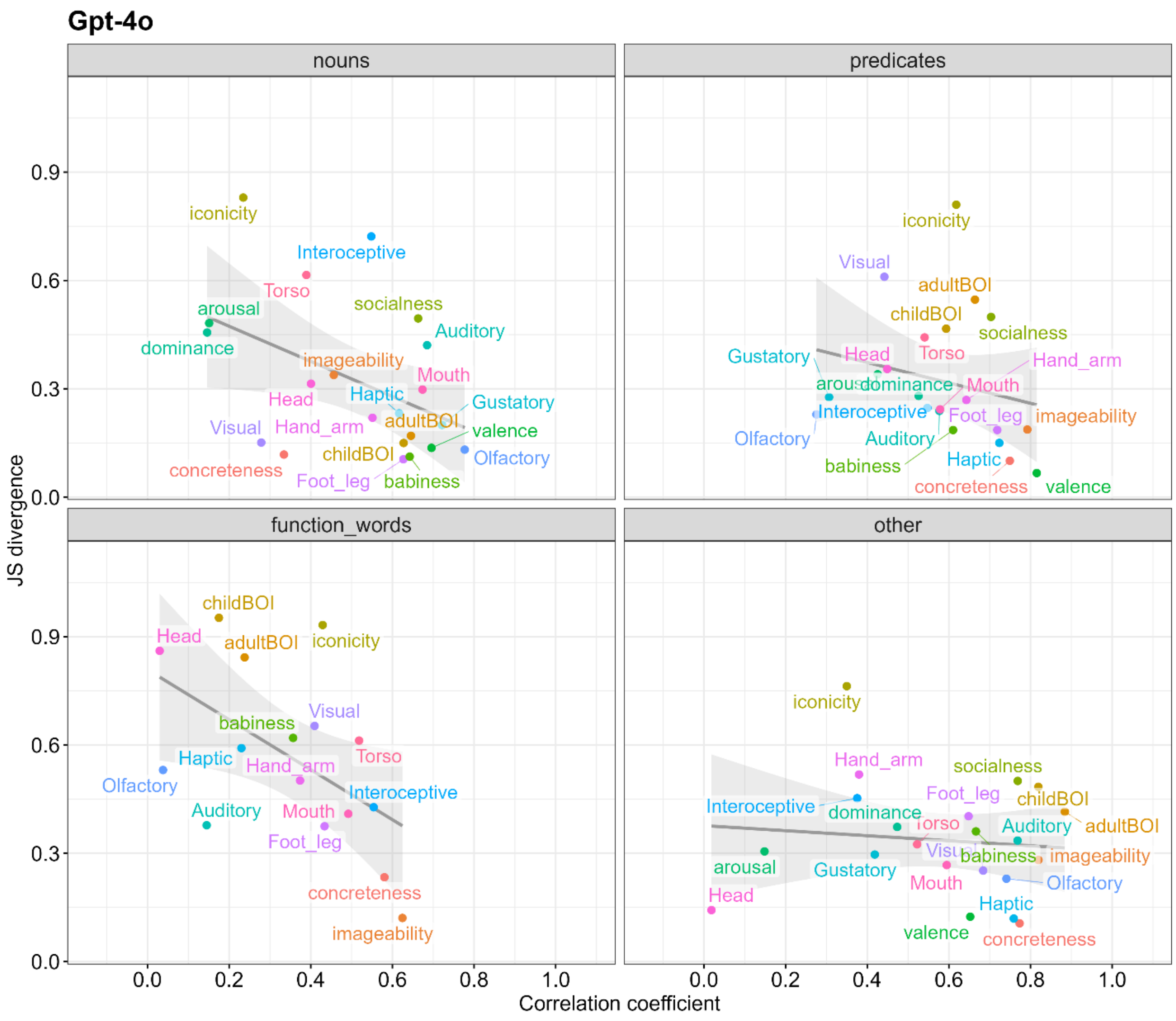

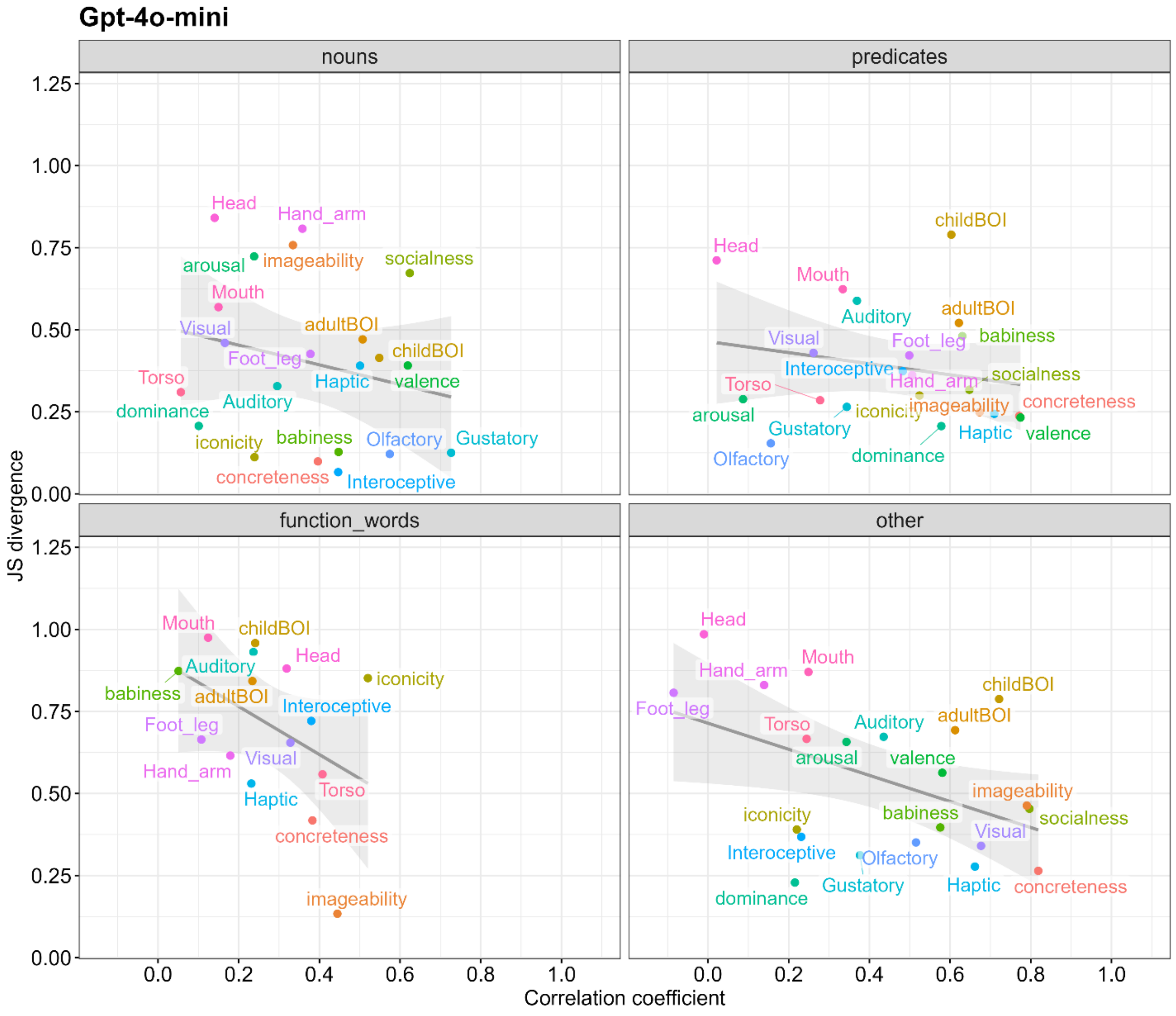
Gpt-4o-mini
nouns
predicates
function_words
other
JS divergence
Correlation coefficient
Head
Hand_arm
arousal
imageability
socialness
Mouth
Visual
adultBOI
Foot_leg
childBOI
Torso
Haptic
valence
Auditory
dominance
iconicity
babiness
Olfactory
Gustatory
concreteness
Interoceptive

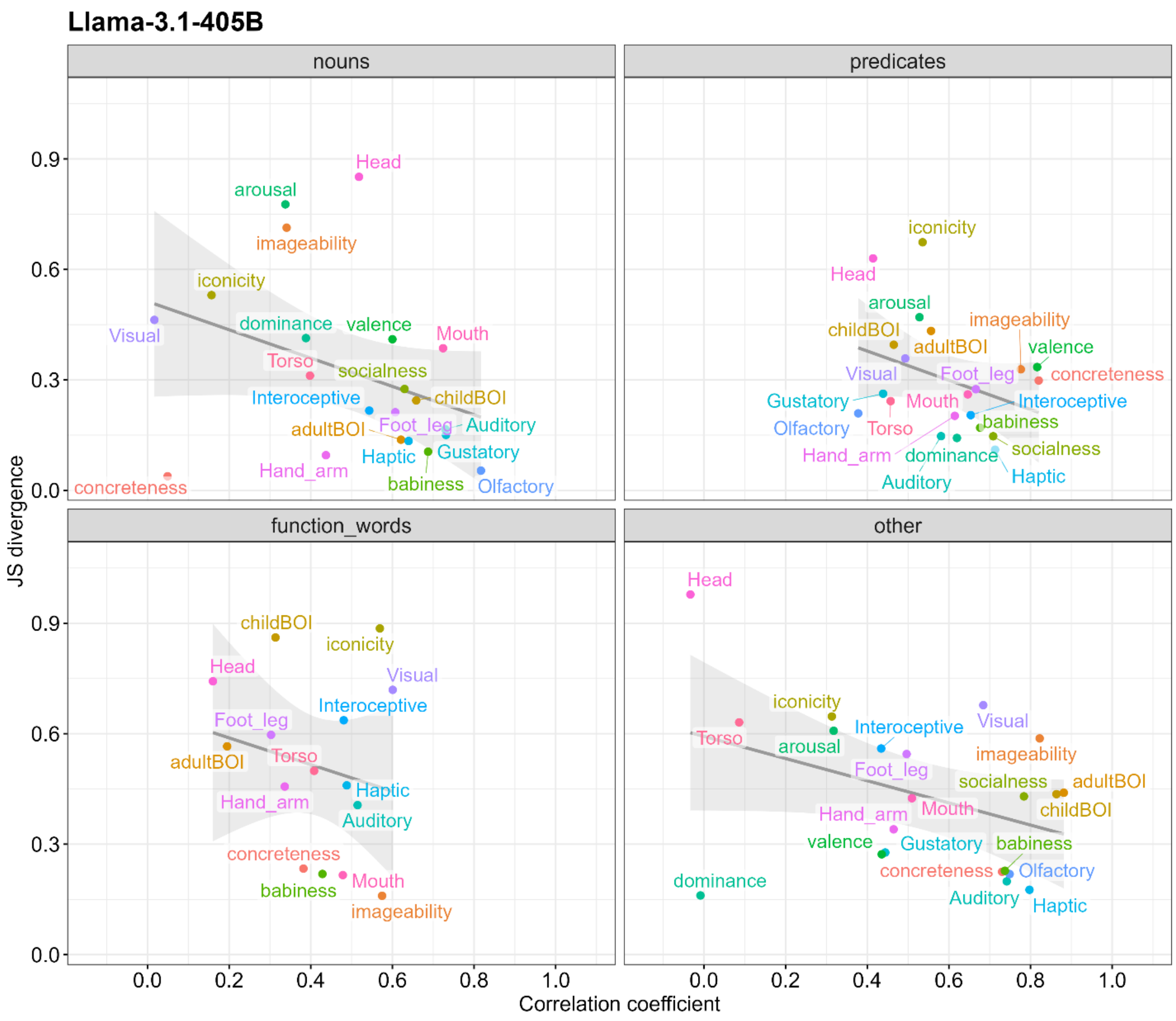
Llama-3.1-405B
nouns
predicates
function_words
other
JS divergence
Correlation coefficient
0.0
0.3
0.6
0.9
0.2
0.4
0.8
1.0
Head
arousal
imageability
iconicity
Visual
dominance
valence
Mouth
Torso
socialness
Interoceptive
childBOI
adultBOI
Foot_leg
Auditory
Haptic
Gustatory
Hand_arm
babiness
Olfactory
concreteness

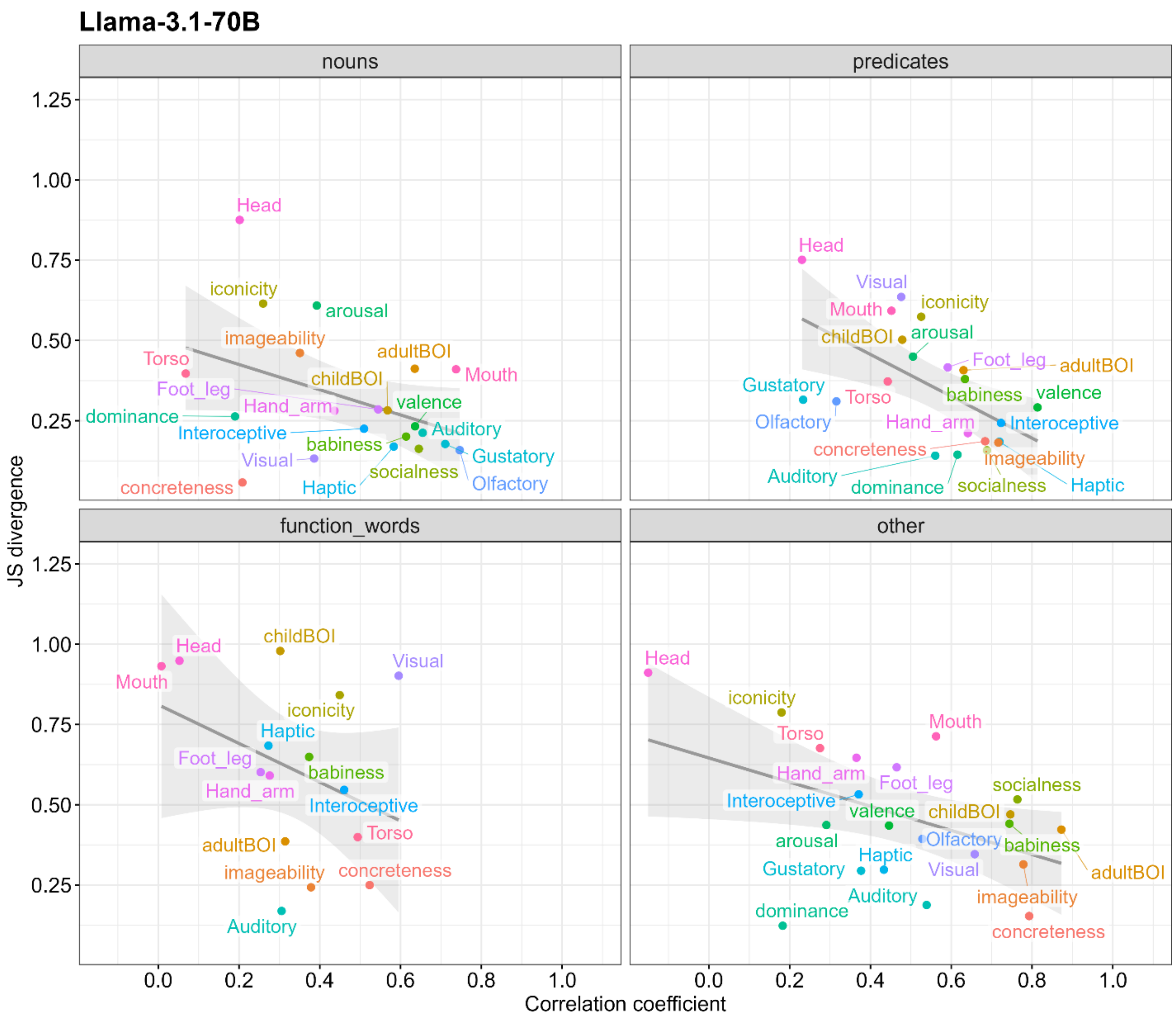

Notes. We excluded Socialness, Valence, Arousal, and Dominance for Function words, because more than 90% of the human ratings were missing (see also Table S2). In addition, for GPT-4o, Gustatory within Function words showed no variance in LLM-derived ratings, preventing the calculation of correlation coefficients. The same issue was observed for both Gustatory and Olfactory within Function words in the other three models. Overall, Function words exhibited relatively larger JS divergences, indicating greater discrepancies from human ratings. See Figure 2 in the main text for details on how to interpret the plots (e.g., legends and symbols).

Figure S3

Predictability of AoA from each psychological feature rated by humans and LLMs while controlling for Concreteness and word frequency

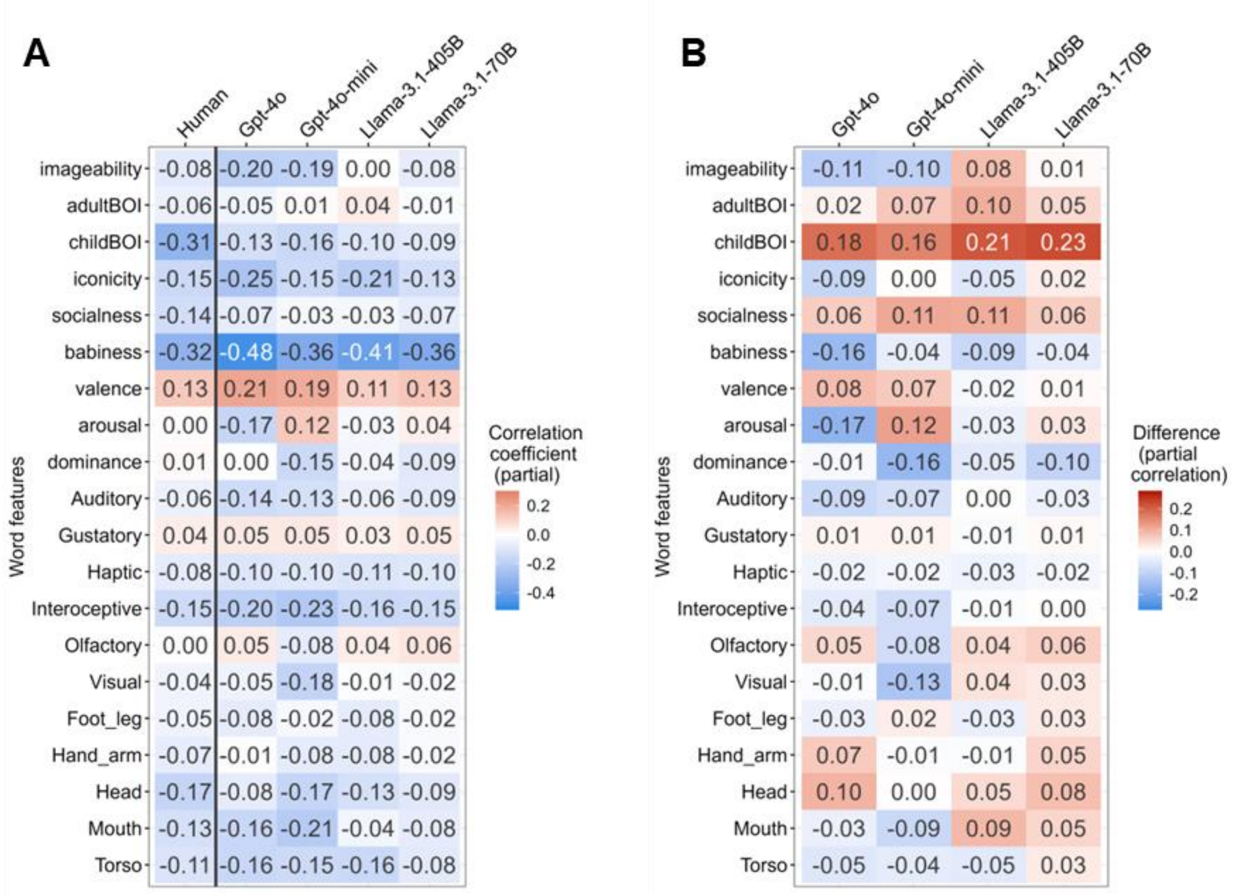

Notes. Spearman's rank correlation coefficients between word AoA and psychological features (A), and the differences in those correlations between humans and LLMs (B), both controlling for Concreteness (human-derived) and word frequency (computed from the CHILDES corpus following the procedure described by Sánchez et al., 2019).

Figure S4

Relationship between partial rank correlation coefficients of AoA and psychological features rated by humans and LLMs, controlling for Concreteness and word frequency

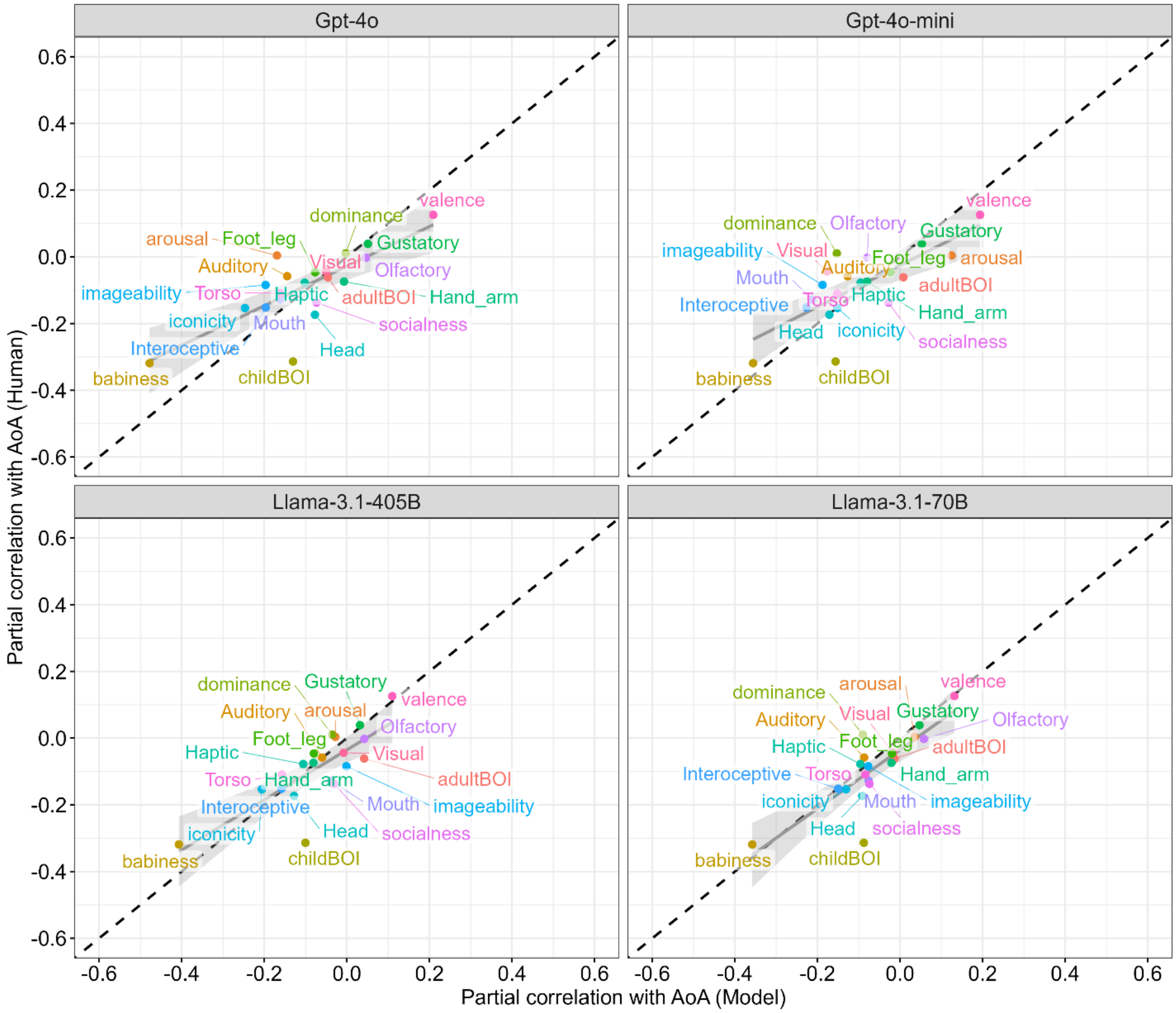

Notes. See **Figure 6** in the main text for details on how to interpret the figure. Similar to the simple correlations (without control variables), both GPT-4o and GPT-4o-mini tended to overestimate the strength of correlations between psychological features and AoA compared with human ratings (*ps*<.010), whereas no significant differences were observed for the other two LLMs (*ps*>.094).

Figure S5

The embedding space of psychological features for each LLM using t-SNE

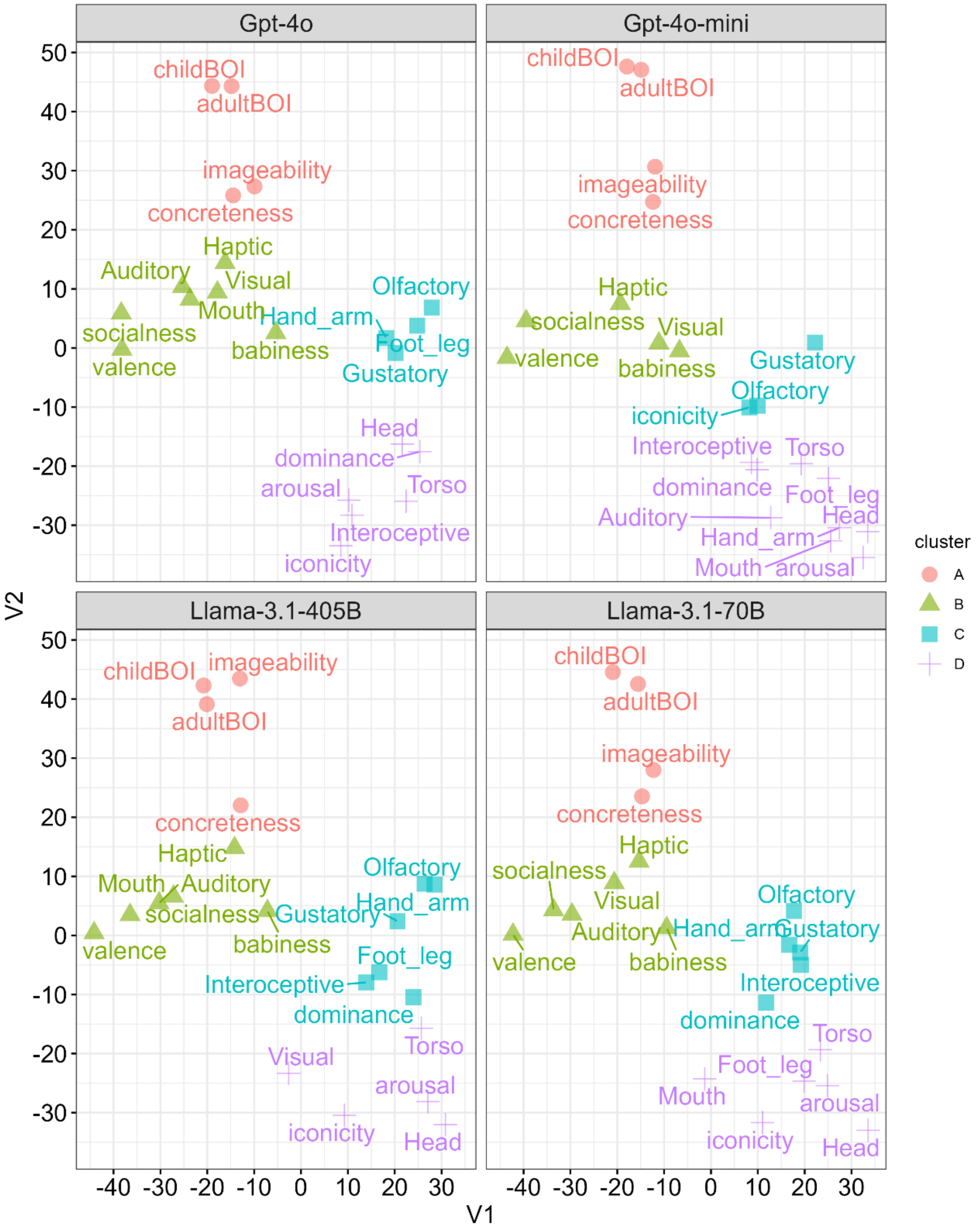

Notes. The k-means approach was applied to clustering.

Figure S6

Results of clustering psychological features in each model based on human–model similarities (UMAP + GMM)

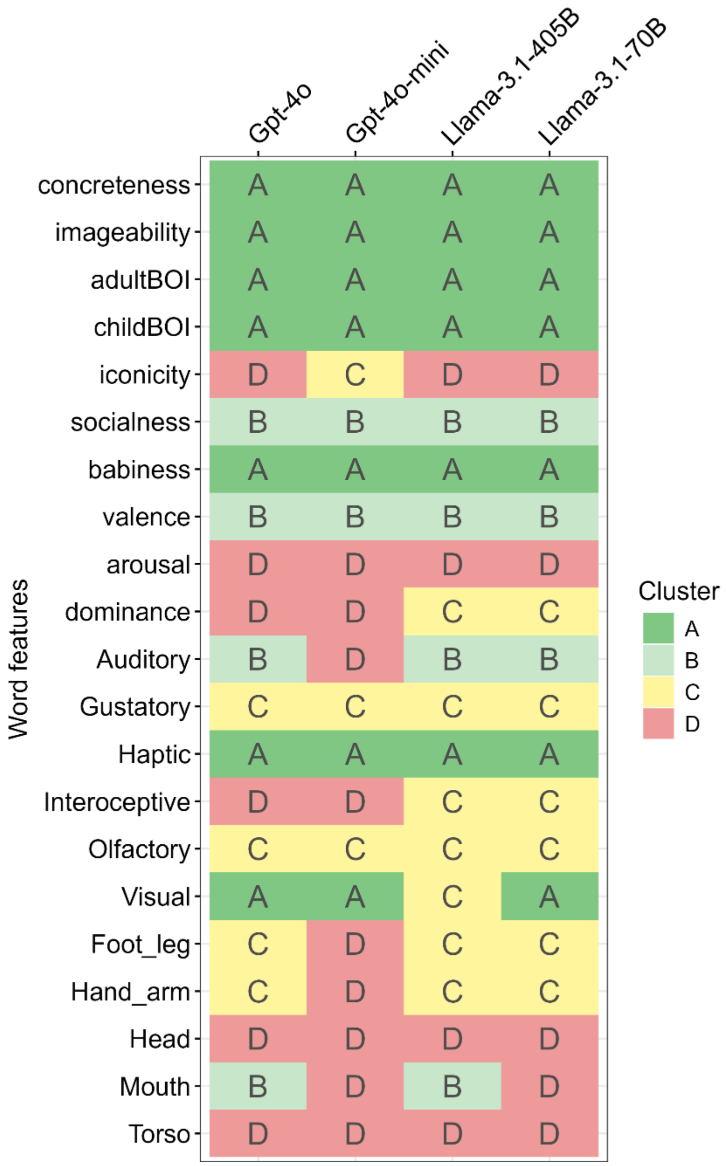

Notes. After dimensionality reduction using UMAP, clustering was performed with a Gaussian mixture model (GMM). The number of clusters was fixed at four, following the procedure described in the main text. The adjusted Rand index (ARI) between this UMAP + GMM solution and the t-SNE + k-means clustering in the main text was .69, indicating reasonable agreement. See **Figure S7** for the interpretation of cluster characteristics.

Figure S7

Mean similarity indices between human and model ratings across clusters (UMAP + GMM)

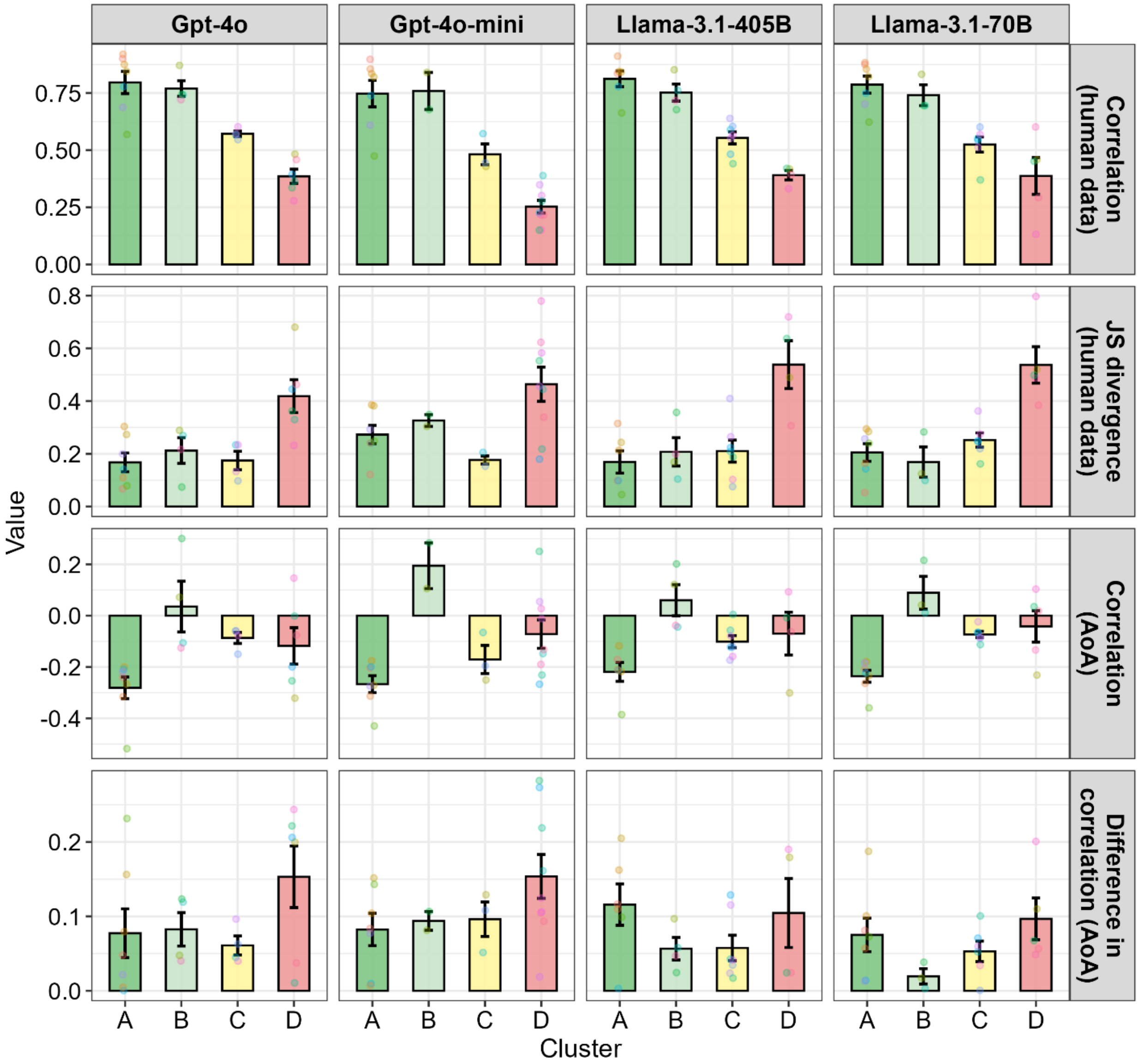

Notes. The cluster characteristics were qualitatively consistent with those presented in **Figure 8** of the main text, confirming that the observed clustering patterns were not artifacts of the t-SNE embedding.

Figure S8

Change in performance of each LLM with different proportions of calibration data

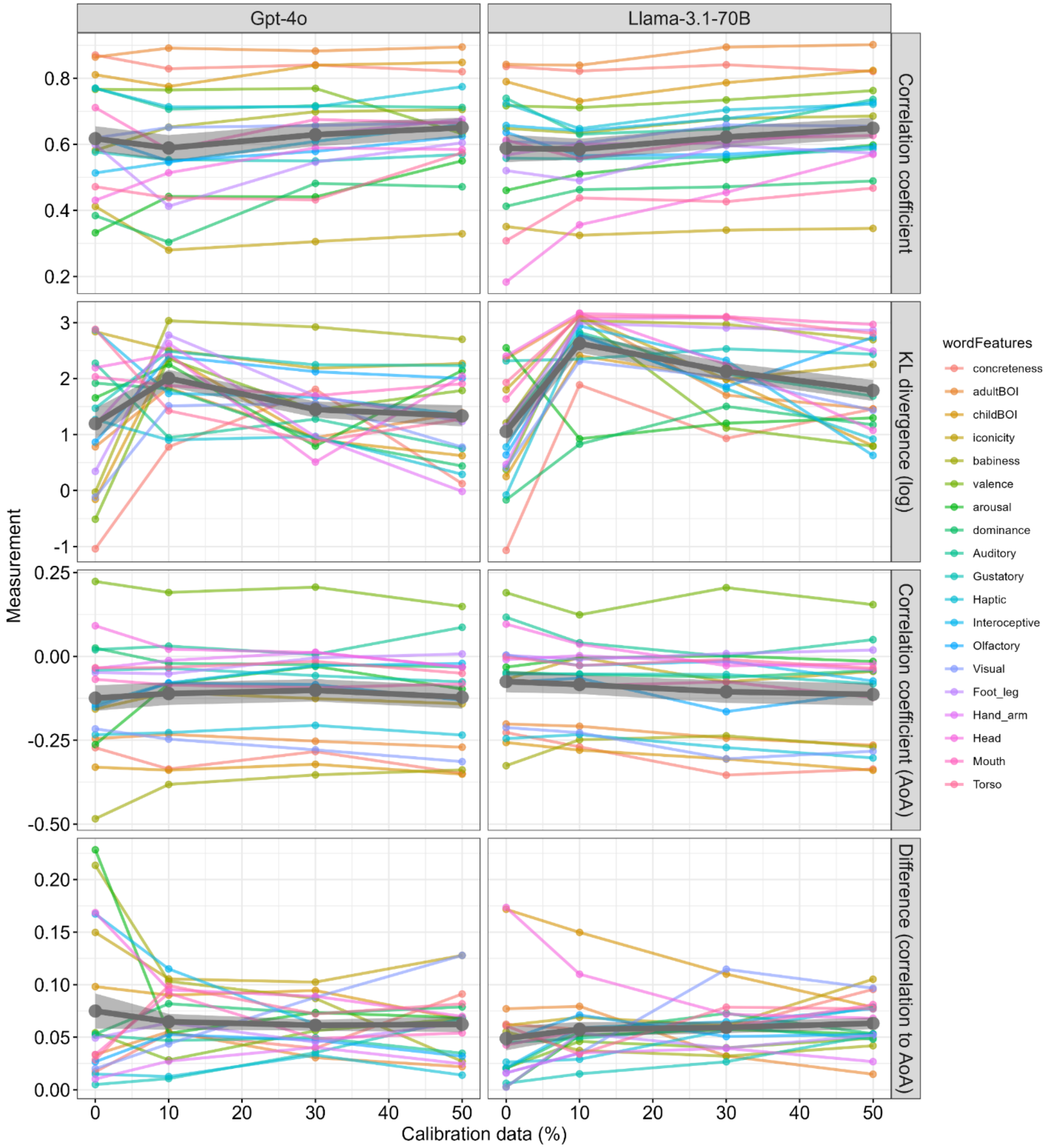

Notes. We conducted a follow-up study to explore the possibility that providing a subset of human ratings for calibration would help LLMs produce psychological feature ratings that are closer to the human judgments of the remaining words. Two features, namely Imageability and Socialness, were excluded because of substantial missing values in the human data (see **Table S2**). The

analysis focused on 428 words for which all remaining psychological features were available. These words were split into calibration and test sets at five levels (10% to 50% for calibration data), while ensuring a balanced distribution of word types (e.g., Nouns and Predicates). However, the results showed no clear improvement in the LLMs' performance with additional calibration data. The thick black lines represent the overall means, and the gray ribbons indicate standard errors when collapsed across features.

Figure S9

Comparison of rating variability between human norms and LLMs

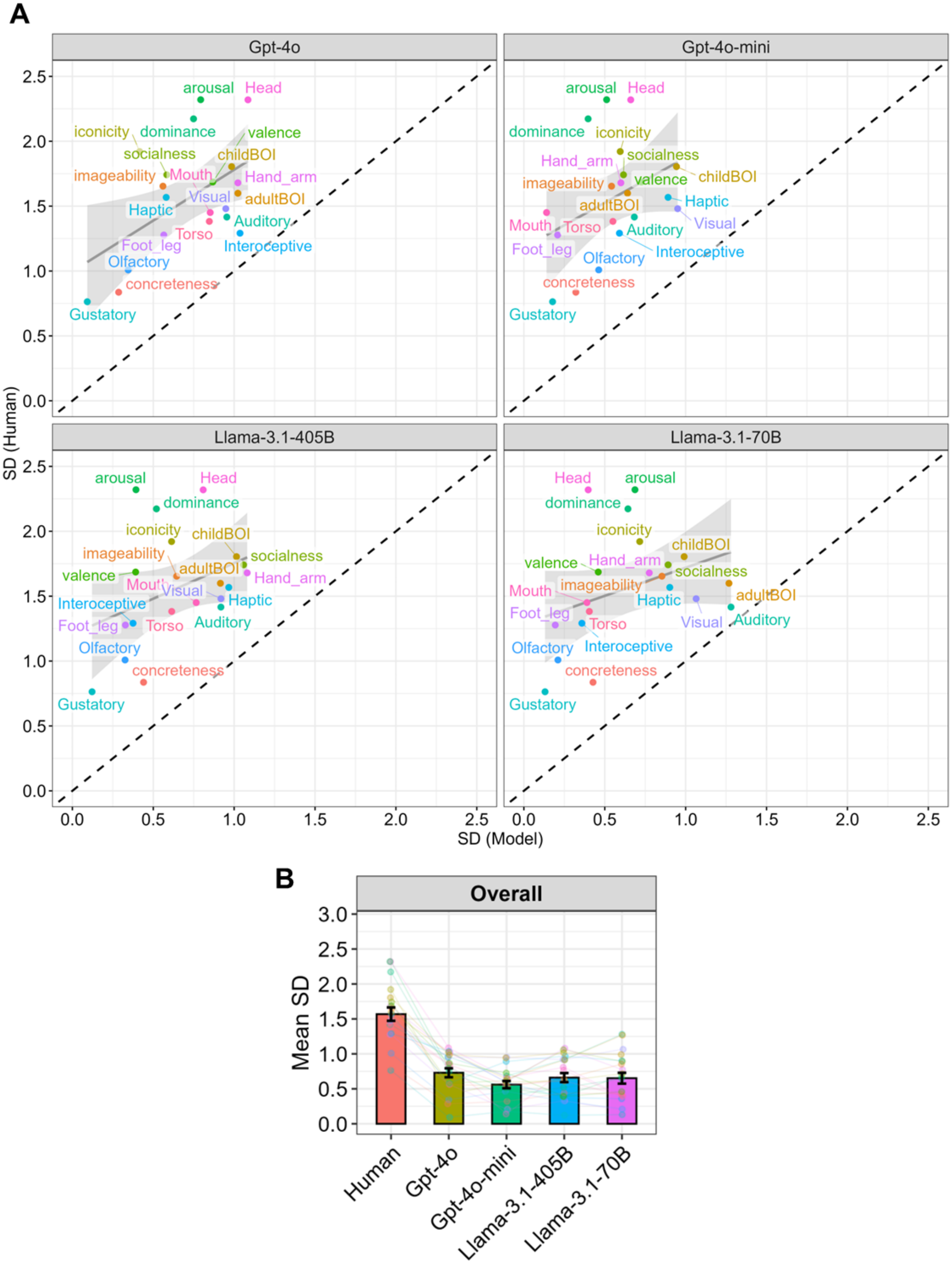

Notes. (A) Scatterplots comparing the mean SDs of rating values between humans and LLMs across 20 psychological features (excluding Babiness, for which the original study did not report SDs). For each target word, the reported SDs from the original studies were used for the human norms, whereas for the LLMs, SDs were calculated across 10 trials. The resulting SDs for each word were then averaged within each psychological feature. The gray lines represent regression lines, and the gray ribbons indicate 95% confidence intervals. All data points lie above the dashed diagonal line, indicating that the variability of LLM-generated ratings was consistently smaller than that of human ratings. (B) Bar plots showing the means and standard errors of SDs averaged across all lexical psychological features. Individual data points (connected by lines) correspond to specific features. A consistent pattern was observed across lexical categories (e.g., Nouns, Predicates), with LLMs exhibiting smaller rating variability than humans.
